# Halotolerant Marine Rhizosphere-Competent Actinobacteria Promote *Salicornia bigelovii* Growth and Seed Production Using Seawater Irrigation

**DOI:** 10.3389/fmicb.2020.00552

**Published:** 2020-04-03

**Authors:** Betty T. Mathew, Yaser Torky, Amr Amin, Abdel-Hamid I. Mourad, Mutamed M. Ayyash, Ali El-Keblawy, Ali Hilal-Alnaqbi, Synan F. AbuQamar, Khaled A. El-Tarabily

**Affiliations:** ^1^Department of Biology, College of Science, United Arab Emirates University, Al-Ain, United Arab Emirates; ^2^Department of Mechanical Engineering, College of Engineering, United Arab Emirates University, Al-Ain, United Arab Emirates; ^3^Department of Food, Nutrition and Health Sciences, College of Food and Agriculture, United Arab Emirates University, Al-Ain, United Arab Emirates; ^4^Department of Applied Biology, University of Sharjah, Sharjah, United Arab Emirates; ^5^Abu Dhabi Polytechnic, Abu Dhabi, United Arab Emirates; ^6^Khalifa Center for Genetic Engineering and Biotechnology, United Arab Emirates University, Al-Ain, United Arab Emirates; ^7^College of Science, Health, Engineering and Education, Murdoch University, Murdoch, WA, Australia

**Keywords:** ACC deaminase, aridland, auxins, biological inoculants, ethylene, plant growth promoting bacteria, polyamines, rhizosphere competency

## Abstract

*Salicornia bigelovii* is a promising halophytic cash crop that grows in seawater of the intertidal zone of the west-north coast of the UAE. This study assess plant growth promoting (PGP) capabilities of halotolerant actinobacteria isolated from rhizosphere of *S. bigelovii* to be used as biological inoculants on seawater-irrigated *S. bigelovii* plants. Under laboratory conditions, a total of 39 actinobacterial strains were isolated, of which 22 were tolerant to high salinity (up to 8% w/v NaCl). These strains were further screened for their abilities to colonize *S. bigelovii* roots *in vitro*; the most promising ones that produced indole-3-acetic acid, polyamines (PA) or 1-aminocyclopropane-1-carboxylic acid (ACC) deaminase (ACCD) were selected for rhizosphere-competency under naturally competitive environment. Three outstanding rhizosphere-competent isolates, *Streptomyces chartreusis* (*Sc*), *S. tritolerans* (*St*), and *S. rochei* (*Sr*) producing auxins, PA and ACCD, respectively, were investigated individually and as consortium (*Sc*/*St*/*Sr*) to determine their effects on the performance of *S. bigelovii* in the greenhouse. Individual applications of strains on seawater-irrigated plants significantly enhanced shoot and root dry biomass by 32.3–56.5% and 42.3–71.9%, respectively, in comparison to non-inoculated plants (control). In addition, plants individually treated with *Sc*, *St* and *Sr* resulted in 46.1, 60.0, and 69.1% increase in seed yield, respectively, when compared to control plants. Thus, the synergetic combination of strains had greater effects on *S. bigelovii* biomass (62.2 and 77.9% increase in shoot and root dry biomass, respectively) and seed yield (79.7% increase), compared to the control treatment. Our results also showed significant (*P* < 0.05) increases in the levels of photosynthetic pigments, endogenous auxins and PA, but a reduction in the levels of ACC in tissues of plants inoculated with *Sc*/*St*/*Sr*. We conclude that the consortium of isolates was the most effective treatment on *S. bigelovii* growth; thus confirmed by principal component and correlation analyses. To this best of our knowledge, this is the first report about halotolerant rhizosphere-competent PGP actinobacteria thriving in saline soils that can potentially contribute to promoting growth and increasing yield of *S. bigelovii*. These halotolerant actinobacterial strains could potentially be exploited as biofertilizers to sustain crop production in arid coastal areas.

## Introduction

Saline water dominates the earth, but the supply of fresh water has always been limited especially in the arid regions of the world. At present, “clean” fresh water scarcity is a major problem in many arid and semi-arid regions in the world, including the United Arab Emirates (UAE) (Odhiambo, 2017). This can be considered a major constraint for sustainable development in agriculture and food production. There is an urgent need to meet the ever-increasing demand for food and agriculture products in such countries with crops that could be irrigated with saline or seawater.

*Salicornia bigelovii*, known as dwarf glasswort, is a succulent halophytic (salt tolerant) plant proves to be a potential candidate to grow in salt marshes and intertidal areas on coasts of many countries (Loconsole et al., 2019) and can be irrigated with seawater (Glenn et al., 1998; Ventura and Sagi, 2013). There is an increasing concern about the use of *S. bigelovii* in human and animal diet as well as in biofuel production worldwide (Bañuelos et al., 2018; Doncato and Costa, 2018). In the UAE, the cultivation of *S. bigelovii* has become profitable. In addition to human consumption and animal feed, oilseeds of *S. bigelovii* can potentially be a source for sustainable production of biodiesel and aviation biofuel (Al-Yamani et al., 2013; Shahid et al., 2013). Consequently, *S. bigelovii* is a main component of biofuel feedstock cultivated with aquaculture and mangrove silviculture in the Seawater Energy and Agriculture System (SEAS) to promote integrated food-energy in desert farms (Ray and Anumakonda, 2011; Ríos, 2014; Sharma et al., 2016).

The impact of microorganisms on plant growth, health, and productivity in extreme environments has been tested under different environmental conditions (Verma et al., 2017). Microorganisms are capable of colonizing soils adjacent to the roots of plants (rhizosphere), above-soil portions of plants (phyllosphere) or living within the tissues of plants (endophytes). Plant growth-promoting rhizobacteria (PGPR) are a group of free living soil bacteria that exert beneficial effects on plants (Hassan et al., 2019). These PGPR have the ability to regulate growth, increase nutrients uptake, enhance tolerance to environmental stress, control pathogenic agents and boost yields in plants (Glick et al., 2007; Kumar et al., 2019). Production of metabolites, such as antibiotics, cell wall degrading enzymes, siderophores or hydrogen cyanide, is among the indirect effects of PGPR to increase plant growth (Bashan et al., 2014; Kumar et al., 2019). PGPR can also promote growth directly through nitrogen (N) fixation, enhancement of plant nutrient uptake, stimulation of transport systems in plants and production of plant growth regulators (PGRs) (Backer et al., 2018). The phytohormones, auxins, cytokinins, gibberellins and ethylene (ET), are examples of PGRs. Other PGRs can be non-naturally occurring synthetic compounds with phytohormone-like activities such as signaling molecules (e.g., reactive oxygen species and nitric oxide), simple ions (e.g., Ca^+2^) and polyamines (PA) (Bashan et al., 2004, 2014; Kumar et al., 2019). The hydrolysis of 1-aminocyclopropane-1-carboxylic acid (ACC) by the enzyme ACC deaminase (ACCD) is a major mechanism utilized by PGPR to lower the levels of ET and to reduce environmental stresses *in planta* (Glick et al., 2007; El-Tarabily et al., 2019). In general, PGPR strains may contribute to PGP by one or more of direct or indirect mechanisms (Backer et al., 2018).

It is well documented that PGPR colonizing rhizosphere have enhanced the growth and relieved environmental stresses in many agricultural crops (Beneduzi et al., 2012). However, research of PGPR in marine ecosystems seems to improve health of halophytes (Bashan et al., 2000; Bashan and Holguin, 2002; El-Tarabily et al., 2020). Few studies have demonstrated the effects of PGPR isolated from the rhizosphere or roots of *Salicornia* on its growth and performance (Ozawa et al., 2007; Jha et al., 2012; Mapelli et al., 2013; Hrynkiewicz et al., 2019). These reports have focused on the direct effect of PGPR on growth promotion of *Salicornia* through enhancing N-fixation. Inoculations of sweater irrigated-*S. bigelovii* with N-fixing and/or phosphorus (P)-solubilizing bacteria have resulted in significant growth promotion (Bashan et al., 2000). When inoculated with *Klebsiella pneumoniae* and *Azospirillum halopraeferens*, *S. bigelovii* showed enhanced agronomic traits (e.g., height and biomass) and biochemical properties (e.g., total contents of proteins and lipids) compared to non-inoculated plants (Rueda-Puente et al., 2004). Interestingly, the halotolerant PGPR isolated from roots of *Salicornia* can perform different PGP activities such as N-fixation, P-solubilization, and production of indole-3-acetic acid (IAA) and ACCD. In addition, these bacteria improved seed germination *in vitro* only (Jha et al., 2012; Mapelli et al., 2013). Under greenhouse conditions, the ACCD-producing endophytic actinobacterium *Micromonospora chalcea* enhanced growth and seed yield of *S. bigelovii* (El-Tarabily et al., 2019). Up-to-date, there is little information about the role of actinobacteria as potential PGP to enhance growth of *Salicornia* under saline conditions in greenhouse/field trials.

In general, root colonization and rhizosphere competency are critical prerequisites for selecting successful PGPR candidate (Rilling et al., 2019; El-Tarabily et al., 2020). Mapelli et al. (2013) have reported that the marine bacteria are capable of colonizing *Salicornia* roots *in vitro*. Many reports have focused on isolating bacterial strains from *Salicornia* rhizosphere (Rueda-Puente et al., 2004; Ozawa et al., 2007; Jha et al., 2012; Figueira et al., 2019). Yet, rhizosphere competence of salt-tolerant marine PGPR to competitively colonize *Salicornia* roots with other microbes is still meager. Recently, the PA-producing actinobacteria, *Actinoplanes deccanensis* and *Streptomyces euryhalinus*, isolated from marine environments promote growth and seed yields of *S. bigelovii* stimulating the endogenous levels of PAs and other PGRs (El-Tarabily et al., 2020).

There is a great interest in utilizing PGPR as bio-inoculants to enhance the growth of halophytic plants (El-Tarabily et al., 2019; Mesa-Marin et al., 2019). In the UAE, the cultivation of *S. bigelovii* for livestock feed, aviation biofuel (jet fuel) and renewable energy purposes has become a top priority for the diversification in the UAE’s economy. The investigation of the halotolerant marine actinobacteria exhibiting PGP features and associated with *S. bigelovii* rhizosphere naturally adapted to cope with extreme saline conditions could most likely promote growth of *S. bigelovii*. Therefore, the objectives of this work were to: (i) isolate halotolerant actinobacteria from *S. bigelovii* rhizosphere soils collected from intertidal zone in the UAE; (ii) determine their tolerance to high salinity and their PGP potential under rhizosphere-competent conditions in naturally competitive saline environment; and (iii) examine the ability of the potent isolates to enhance growth of *S. bigelovii* under greenhouse conditions. In this study, plant growth, photosynthetic pigments and levels of endogenous auxins, PA and ACC were evaluated in shoots and roots, in addition to seed yields. Results obtained from the principal component analysis (PCA) support the accuracy of the experiment and the equivalent reliability in screening for plant growth promotion using rhizosphere-competent PGP actionobacteria. The best performing isolates selected were applied individually and in combination to determine whether growth promotion could be enhanced by mixing actinobacterial species showing different mechanisms of action *in vivo*.

## Materials and Methods

### Plant Material and Soil Characteristics

In all experiments, wild-type seeds of *S. bigelovii* Torr. (Scrops, Ninove, Belgium) were surface sterilized by 70% ethyl alcohol (EtOH; Sigma-Aldrich Chemie GmbH, Germany) and 1.05% Clorox (20% household bleach). Two drops of Tween 20 (Sigma-Aldrich) was used in all surface-sterilization steps. Surface-sterilized seeds were then washed with 0.22 μm membrane filter-sterilized (Millipore Corporation, MA, United States) full strength seawater (salinity of 4.0%), and were air-dried before use.

Pale grayish-yellow sandy soil was collected from Al Rams coast, Ras Al Khaimah, UAE (25° 52′ 44″ N, 56° 1′ 25″ E), where *S. bigelovii* is naturally growing. Soil was passed through a 1 cm mesh sieve for all greenhouse experiments. The following soil chemical characteristics were detected: electrical conductivity 9.31 dSm^–1^; pH 8.18 (in 0.01 M CaCl_2_), organic C 1.15%, and the nutrients (mg kg soil^–1^) [N (5.3 as NH_4_^+^; 2.9 as NO_3_^–^), oxalate extractable amorphous Fe (382), sulfate (311), bicarbonate extractable K (265), total P and available P (44, 8.3, respectively)].

### Collection of Rhizosphere Soils

Rhizosphere soils of six young *S. bigelovii* seedlings were collected from the above-mentioned study site. Excavated soil (maximum depth of 15 cm) from around the root balls was placed into sterile plastic bags and brought to the laboratory. Samples were passed through 5 mm sieve of which each sample consisted of rhizosphere soils from two seedlings, randomly pooled as one replicate. The three collected rhizosphere soil samples were air-dried at 25°C for 4 days to reduce the viable vegetative bacterial cells (Williams et al., 1972).

### Isolation of Actinobacteria From *Salicornia* Rhizosphere Soils

The aerobic culturable actinobacterial populations of the freshly sampled rhizosphere soils were determined using the soil dilution plate count method. Three replicates of 10 g rhizosphere soil samples were dispensed into 100 mL of membrane filter-sterilized full strength seawater, and the soil suspension was placed in an ultra-sonic cleaner at a frequency of 55,000 cycles s^–1^ for 20 s. The soil suspension was then shaken at 250 rpm at 28°C for 30 min on a rotary shaker, Model G76 (New Brunswick Scientific, NJ, United States).

Dilutions of 10^–2^–10^–5^ were made in membrane filter-sterilized full strength seawater and 0.2 mL aliquots were spread onto inorganic salt starch agar (ISSA) (Küster, 1959) made with membrane filter-sterilized full strength seawater in sterile petri dishes. Cooled (45°C) sterile ISSA medium was amended with 50 μg mL^–1^ cycloheximide (Sigma-Aldrich) and 50 μg mL^–1^ nystatin (Sigma-Aldrich). Five plates per dilution were used and air-dried in a laminar flow.

Plates were incubated for 7 days at 28°C in dark and colonies were counted (log_10_ colony forming units (cfu) g^–1^ dry soil). Unless otherwise indicated, actinobacteria were purified and maintained on ISP3 medium (oatmeal agar) plates made with membrane filter-sterilized full strength sweater and supplied with 0.1% yeast extract (OMYEA) (Shirling and Gottlieb, 1966). Streptomycete actinobacteria (SA) and non-streptomycete actinobacteria (NSA) were tentatively identified to the genus level according to Cross (1989). Colonies of SA and NSA were identified based on morphological characteristics, ability to form sporangia, distribution of aerial and/or substrate mycelia, presence or absence of aerial mycelia, the stability and fragmentation of substrate mycelia (Cross, 1989). Isolates were stored as mycelia and spores in 20% glycerol at −20°C (Wellington and Williams, 1977).

### Tolerance of Actinobacterial Isolates to Different Concentrations of NaCl

Tolerance to salt stress was evaluated by growing the selected isolates on ISSA medium made with deionized water and supplemented with the following NaCl concentrations: 0, 10, 20, 40, 60, and 80 g L^–1^ medium. The isolates were streaked in triplicates on the plates and incubated for 7 days at 28°C in dark (Williams et al., 1972). Actinobacterial growth and heavy sporulation on ISSA supplemented with 80 g NaCl L^1^ (8%) indicated the efficiency of the selected isolates to tolerate high NaCl concentration and to be true halotolerant isolates. Only these isolates were chosen for subsequent experiments.

### *In vitro* Indicator Root Colonization Plate Assay

The ability of the obtained halotolerant actinobacterial isolates to colonize S. *bigelovii* was carried *in vitro* using the primary qualitative root colonization plate assay (Kortemaa et al., 1994) to determine if *S. bigelovii* root exudates can act as the only carbon source to support the growth of each isolate. Surface-sterilized *S. bigelovii* seeds were pre-germinated on membrane filter-sterilized full strength seawater agar plates for 2 days before being transferred onto new plates (one seed/plate). Eight replicates were inoculated with each isolate grown on OMYEA (one isolate/plate). Seeded plates without treatment were used as controls. Actinobacterial suspension (approximately 10^8^ cfu mL^–1^) was prepared for each isolate from OMYEA plates made with membrane filter-sterilized full strength seawater. An inoculum (0.2 mL) was placed 1–2 mm alongside the emerging radicle (Kortemaa et al., 1994). The seawater agar plates were vertically incubated at 28°C in dark and the root colonization by the isolates was detected after 8 days. Colonization was calculated as a percentage of the total root length (Kortemaa et al., 1994).

### Scanning Electron Microscopy (SEM)

According to the indicator root colonization plate assay, roots were initially fixed with Karowskys fixative (2.5% glutaraldehyde and 2% para-formaldehyde) for 4 h at 25°C. Samples were treated with 0.2 M phosphate buffer, pH 7.2 at 4°C for 2 h and post-fixed in 1% osmium tetroxide for 1–2 h. After washing with deionized water, samples were dehydrated with a series of EtOH dilutions ranging from 30 to 100% at 4°C. Once in 100% EtOH, roots were dried in a critical-point dryer (Polaron CPD Bell Brook Business Park, Bolton Close England) and mounted on carbon-tabbed aluminum (Al) stubs. Stubs were fixed in the Polaron sputter coater vacuum chamber and sputtered with gold Au/Pd target for 5 min at 20 mA. Phillips XL-30 SEM (Eindhoven, The Netherlands) was used to examine the colonized root samples.

### Production of IAA, PA, and ACCD by Actinobacteria

All microbiological media in the experiments described in sections Production of IAA, PA, and ACCD by Actinobacteria and Other PGP Activities of the Three Promising Isolates were prepared using membrane filter-sterilized full strength seawater. The selected root colonizing isolates were *in vitro* tested for their capability to produce IAA, PA, and ACCD. To detect IAA, Erlenmeyer flasks (100 mL) containing 50 mL of sterile inorganic salt starch broth (ISSB) (Küster, 1959) were supplied with 5 mL of 5% filter-sterilized L-tryptophan (Khalid et al., 2004). The flasks were inoculated with 2 mL of each isolate prepared from a 5 days old shaken ISSB culture of about 1 × 10^8^ cfu mL^–1^, covered with Al-foil and incubated on a rotary shaker (Model G76) at 250 rpm at 28°C in dark for 7 days. Non-inoculated flasks were used as a control. After incubation, suspensions from each flask were centrifuged at 12,000 × *g* for 30 min. The supernatant was filtered through Millipore membranes and collected in sterile tubes. Six milliliters of culture supernatants were pipetted into test tubes and 4 mL of Salkowski reagent (2 mL of 0.5 M FeCl_3_ + 98 mL 35% HClO_4_) were applied (Gordon and Weber, 1951). Tubes were left for 30 min to develop red color; and the intensity of the color was determined by optical density at 530 nm using a scanning spectrophotometer (UV-2101/3101 PC; Shimadzu Corporation, Analytical Instruments Division, Kyoto, Japan). A standard curve was established according to the color of the standard solutions of IAA, and auxin compounds were expressed as IAA-equivalents (Gordon and Weber, 1951). Eight replicate samples were analyzed.

The obtained actinobacterial isolates were tested for the production of putrescine (Put) as a polyamine using Moeller’s decarboxylase agar medium (MDAM) amended with 2 g L^–1^ L-arginine-monohydrochloride (Sigma-Aldrich) and 0.02 g L^–1^ phenol red (Sigma-Aldrich) (Arena and Manca de Nadra, 2001). Isolates growing on OMYEA were streaked in triplicate on MDAM plates with or without Arginine (control). Plates were incubated at 28°C for 3 days in dark. Production of Put by the decarboxylating isolates was determined by the existence of a dark red halo beneath and around the colonies.

All isolates showing large red halo color on MDAM were further tested for production of Put, spermidine (Spd), and spermine (Spm) in Moeller’s decarboxylase broth medium (MDBM) amended with 2 g L^–1^ of L-arginine-monohydrochloride by reverse-phase high-performance liquid chromatography (HPLC; SpectraLab Scientific Inc., ON, Canada). Erlenmeyer flasks containing 50 mL of MDBM were inoculated with 3 mL of 20% glycerol suspension of each isolate (10^8^ cfu mL^–1^) and incubated on a rotary shaker at 250 rpm at 28°C. After incubation for 10 days in dark, the suspension from each flask was centrifuged at 12,000 × *g* for 30 min. The membrane filter-sterilized cell-free culture broth was collected in sterilized McCartney tubes.

The derivatization of the filter-sterilized cell-free filtrates and the internal polyamine standards were carried out as follows: 0.5 mL of filtrate was added to 0.5 mL of saturated solution of sodium bicarbonate (Sigma-Aldrich) and 2 mL of dansyl chloride (Sigma-Aldrich) solution (10 mg mL^–1^ in acetone). The reaction vessel was incubated at 60°C for 20 min and 50 μL of 28% ammonium hydroxide were added. After 30 min, the reaction mixture was filtered again through 0.22 μm Millipore membranes and used for the HPLC (Marino et al., 2000). The HPLC chromatograms (eight replicates of each isolate) were produced by injecting 10 μL aliquot of the sample onto a 10 μm μBondapak C_18_ column (4 mm × 30 cm) in liquid chromatograph (Waters Associates) equipped with a 254 nm UV detector (Smith and Davies, 1985).

The selected isolates were also screened for the production of ACCD. Five-day-old isolates grown on rich OMYEA were streaked in triplicates on N-free Dworkin and Foster’s salts minimal agar medium (DF) plates (Dworkin and Foster, 1958) amended with either 3 mM ACC (Sigma-Aldrich) or 2 g (NH_4_)_2_SO_4_ (control). The heat-labile ACC was filter-sterilized and the filtrate was added to the salt medium. Plates were incubated at 28°C in dark for 7 days. Isolates growing on DF + ACC plates indicated the efficiency of the isolate to utilize ACC and produce ACCD. To determine the activity of ACCD, isolates were grown in ISSB at 28°C for 5 days in dark. Cells were centrifuged, washed with 0.1 M Tris-HCl (pH 8.5) and inoculated onto DF + ACC broth on a rotary shaker at 250 rpm for another 5 days. Cells were resuspended in 0.1 M Tris-HCl and ruptured by freezing/thawing cycles thrice (Shah et al., 1998). The lysate was centrifuged at 80,000 × *g* for 1 h. The enzymatic activity of ACCD was assayed by monitoring the amount of α-ketobutyrate liberated (Honma and Shimomura, 1978). Protein concentrations were determined as described (Bradford, 1976). Eight replicate samples were analyzed.

### Rhizosphere Competence Assay Under Naturally Competitive Environment

To investigate whether particular members of actinobacteria producing high levels of IAA, PA, or ACCD are avid colonizers of the rhizosphere (rhizosphere-competent), the non-sterilized soil tube assay was used (Ahmad and Baker, 1987) using rifampicin resistant mutants (Misaghi and Donndelinger, 1990).

The surface-sterilized *S. bigelovii* seeds were coated with each isolate as described below. Non-treated seeds served as controls. In each tube containing non-sterilized field soil (collected from the same site described above), one seed was planted 5 mm deep. Tubes were vertically placed into boxes containing the same soil and watered with full strength seawater to container capacity. Boxes were covered and held upright with no more added seawater to the tubes after sowing (Ahmad and Baker, 1987). Boxes were kept in a greenhouse (photosynthetic photon flux density of 700 μmol m^–2^ s^–1^) at 25 ± 2°C and relative humidity (RH) of 60 ± 5%.

After 4 weeks, *S. bigelovii* were harvested from the soil tube assay. The length of the harvested roots was measured, and only the top 12 cm of roots were retained, and were aseptically cut into 2 cm segments (Ahmad and Baker, 1987). Loose particles of the rhizosphere soil on root segments were removed and air-dried. Rhizosphere soil particles were numbered based on the root segments from which they were recovered (Ahmad and Baker, 1987). Roots and soil particles were separately studied to determine the root-colonization frequencies (R%) and population densities (PD; log_10_ cfu g^–1^ dry soil) of each isolate in the rhizosphere soil, respectively (Ahmad and Baker, 1987) on ISSA plates made with membrane filter-sterilized full strength sweater and supplemented with 100 μg mL^–1^ rifampicin (Sigma-Aldrich). To be considered as colonized-rhizosphere or -root segment, colonies of the isolated strains were obtained on ISSA plates amended with rifampicin either in the corresponding rhizosphere soil sample, on the root segment or both (Ahmad and Baker, 1987) after 7 days of incubation at 28°C in dark. Percentage of root segments colonized was calculated as the proportion of the total number of segments colonized to the total segments sampled at each distance. Experiments were repeated twice with eight replicates in each; and only the strongest three rhizosphere-competent isolates producing either IAA, PA, or ACCD were chosen for the greenhouse experiments.

### Identification and Construction of Phylogenetic Tree of Selected Actinobacteria

Amplification of 16S rRNA gene using polymerase chain reaction (PCR) of the three isolates (#7, #1, and #21) and their sequencing were carried out by Deutsche Sammlung von Mikroorganismen und Zellkulturen GmbH (DSMZ, Braunschweig, Germany). According to Rainey et al. (1996), the PCR primers used were: 900R (5′-CCGTCAATTCATTTGAGTTT-3′); 357F (5′-TACGGGAGGCAGCAG-3′) and 800F (5′-ATTAGATACCCTGGTAG-3′). Nucleotide sequences were submitted to GenBank and assigned accession numbers MN795132, MN795133, and MN795131 for isolates #7, #11, and #21, respectively.

Pairwise sequence similarity using 16S rRNA gene sequence was determined. For the phylogenetic analyses, reference strains were selected from the top hits of the determination using GenBank BLAST^1^. An amplification of 1,516, 1,519, and 1,519 bp from isolates #7, #11, and #21 was obtained and aligned with closely-related strain sequences of *Streptomyces* using CLUSTAL-X (Thompson et al., 1997) in Molecular Evolutionary Genetics Analysis 7.0 (MEGA7) software (Kumar et al., 2016). Phylogenetic trees were constructed using the maximum likelihood (ML) method of which bootstrap values were calculated based on 1000 resamplings. The morphology of the spore chains and surface was carried out for the three isolates using Phillips XL-30 SEM.

### Other PGP Activities of the Three Promising Isolates

To detect auxins as IAA and IPYA (indole-3-pyruvic acid), the three isolates (#7, #11, and #21) were cultivated in 50 mL ISSB supplemented with 5 mL of 5% L-tryptophan (Sigma-Aldrich). Gibberellic acid (GA_3_) and cytokinins [isopentenyl adenine (iPa), isopentenyl adenoside (iPA) and zeatin (Z)], were detected on Strzelczyk and Pokojska-Burdziej (1984) medium. After 10 days, the extraction of these PGRs from the concentrated filter-sterilized cell-free broth and the HPLC parameters used to determine the concentrations of IAA, IPYA, GA_3_, iPa, iPA, and Z were carried out as described by Tien et al. (1979).

The production of siderophores was determined using chrome azurol S agar plates (Schwyn and Neilands, 1987). Yellow-orange halo zone developed around the colony was considered positive for siderophores production. The N-fixing activities of isolates were examined by the acetylene reduction assay (Holguin et al., 1992) using Varian 6000 gas chromatogram (Varian Instrument Group, United States). Production of NH_3_ was determined using Nessler’s reagent (Dye, 1962). Solubilization of insoluble rock phosphate (Tianjin Crown Champion International Co., Ltd., Tianjin, China) was estimated using Pikovskaya’s agar medium (Pikovskaya, 1948) with bromophenol blue in which tricalcium phosphate was replaced with rock phosphate. Production of clear zone was an indicator of P-solubilization. All rock phosphate-solubilizing actinobacterial isolates showing large zones of clearing on Pikovskaya’s agar were further tested quantitatively for their abilities to solubilize rock phosphate in modified National Botanical Research Institute’s phosphate broth (Nautiyal, 1997) containing 5 g L^–1^ rock phosphate. Water-soluble P was analyzed by the colorimetric procedure of Murphy and Riley (1962) using molybdophosphoric acid blue complex. The drop in pH and the released soluble P amount were taken as an indicator of the efficiency of isolates. In all the above-mentioned tests, eight independent replicates were used for each isolate.

### Assessment of Inhibitory Activity Among Actinobacterial Isolates

The spot-inoculated plate technique described by de Boer et al. (1999) was used to determine the isolate’s sensitivity to the diffusible metabolites of the other isolates. Suspensions of actinobacterial spores were cultured on OMYEA in 10 mM MgSO_4_. For each isolate, approximately 10^8^ cfu mL**^–^**^1^ was used as an inoculum. Actinobacterial isolates were spot-inoculated on OMYEA by pipetting 2 droplets of 5 μL of the actinobacterial inoculum; and plates were incubated at 28°C for 5 days in dark. Later on, a suspension of the target isolate was atomized over the surface of the spot-inoculated plates. After 4 days at 28°C in dark, zones of growth inhibition of the target isolate around the spot-inoculated isolates were evaluated, if any.

To determine if the selected isolates do not inhibit each other by their volatile compounds, the method described by Payne et al. (2000) was used. Briefly, fresh OMYEA plates were inoculated by evenly spreading 0.2 mL of 10^8^ cfu mL**^–^**^1^ of the isolate onto the surface of agar. Plates were incubated in dark at 28°C. After 5 days of incubation, another fresh plate of OMYEA was simultaneously inoculated with an actively growing culture of the tested isolate. The lids were removed and the plates containing the tested isolate were inverted over the previously grown isolate; and the two plate bases were taped together with Parafilm (American National Can TM, Greenwich, CT, United States). Control plates were prepared the same way except that non-inoculated plate was used instead of a plate containing the target isolate. After another 5 days in incubator, the growth of the target isolate was compared to that of the control. In these two tests, four independent replicates were used for each isolate.

### Preparation of Date Pits-Based Activated Carbon as Seed Adsorbents for Actinobacterial Isolates

For the preparation of activated carbon as seed adsorbents for the actinobacterial isolates to be used in greenhouse experiments, local date seeds from the fruits of date palm (*Phoenix dactylifera*) were dried and ground using an electric agitated mortar (JK-G-250B2, Shanghi Jingke Scientific Instrument) (Mathew et al., 2018). Physical activation of date seeds was performed in a tube furnace (GSL-1500X; MTI Corporation, VA, United States) with carbonization followed by activation. A carbonization step was started by allowing N_2_ gas to pass through the furnace for 10 min when the temperature was gradually increased under a constant flow of N_2_ at a rate of 5°C min^–1^ up to 600°C and maintained at this temperature for 4 h (Sekirifa et al., 2013). The carbonaceous material was activated at 900°C in the same furnace under the flow of CO_2_ gas. The activated carbon was then sieved using a testing mesh (size of 200–300 μm). To prepare the activated carbon nanomaterials, activated carbon was wet-ground in a grinder (Retsch RM 100, Germany) and dried in a freeze dryer (Telstar, Spain) at −55°C and 0.02 mbar for 6 h (Mathew et al., 2018). The material was then filtered using a 0.45 μm polytetrafluoroethylene filter (Thomas Scientific, NJ, United States) prior to use as a seed adsorbent for actinobacterial isolates.

### Production of Inocula for *in vivo* Experiments

Inocula of each isolate were applied as seed coating and in soil for the greenhouse experiment described below as recommended by El-Tarabily (2008).

For the seed coating, isolates grown in ISSB made with membrane filter-sterilized full strength sweater were shaken at 250 rpm at 28°C for 5 days in dark. Cells and/or spores were collected and centrifuged for 20 min at 5,000 × *g*. Pellets were washed three times before being suspended in sterile 0.03 M MgSO_4_ (buffer). Instantly, surface-sterilized *S. bigelovii* seeds were immersed in beakers containing either each actinobacterial suspension (approximately 10^8^ cfu mL^–1^) in 0.03 M MgSO_4_ (buffer) or buffer alone (no actinobacterial suspension; control) for 4 h at 28°C. Activated carbon from date seeds (5 g L^–1^) was added as an adsorbent for the treated and the control seeds. The rest of the suspension was drained and seeds were air-dried.

For each isolate, inocula were prepared for the soil application by adding 300 g of oat bran as a food source in Erlenmeyer flasks (500 mL) autoclaved for 20 min at 121°C on three successive days. The oat bran was then aseptically inoculated with spore suspensions (25 mL) of each isolate in 20% glycerol (10^8^ cfu mL^–1^) and incubated in dark at 28°C for 3 weeks. Non-colonized oat bran, which had been autoclaved twice, served as a control. Colonized oat bran with each isolate (0.05 g colonized oat bran inoculum g^–1^ air-dried non-sterile soil) were mixed thoroughly using a cement mixer in all experiments described below.

### Evaluation of Growth Promotion of *S. bigelovii* in the Greenhouse

*S. bigelovii* growth was *in vivo* tested to determine the effect of the three isolates #7, #11, and #21 either individually or combined three together under greenhouse conditions. Briefly, free draining plastic pots (23 cm in diameter) were filled with 7 kg soil sampled from the area (described above) and mixed with 0.05 g of colonized oat bran inoculum g^–1^ field soil. Surface-sterilized healthy *S. bigelovii* seeds were coated with each isolate and with the activated carbon from date pits (described above) or with activated carbon only (control). In each pot, eight seeds were sown in soil at 5 mm deep, and seedlings were thinned to four/pot when emergence was complete. A total of five treatments were applied; where each treatment was replicated eight times with four seedlings/replicate as follows: Non-inoculated control (C); individually inoculated with either isolate #21 (*Sc*), isolate #7 (*St*) or isolate #11 (*Sr*), producing auxins, PA or ACCD, respectively; and collectively inoculated with all three isolates (*Sc*/*St*/*Sr*).

Pots were placed in the greenhouse (photosynthetic photon flux density of 700 μmol m^–2^ s^–1^) at 25 ± 2°C and RH of 60 ± 5%) in a randomized complete block design (RCBD), and were daily watered to container capacity with full strength seawater. Dry weight and length of shoots and roots were recorded at 12 weeks post-sowing (wps) the seeds, and seed dry weight was recorded 20 wps.

### Extraction of Photosynthetic Pigments, Endogenous Auxins, PA, and ACC From *S. bigelovii*

The levels of photosynthetic pigments, including chlorophyll (chl *a* and chl *b*) and carotenoids, were determined in the succulent stems (Holden, 1965; Davies, 1965). Tissues of the terminal part of the root and shoot systems were used for the analysis of endogenous auxins, PA, and ACC. The free PA (Put, Spd, and Spm) were extracted according to Flores and Galston (1982). Benzoylation using benzoyl chloride (Sigma-Aldrich) and the HPLC for the plant extracts and the internal standard of PA were carried out as described by Redmond and Tseng (1979). The extraction of endogenous IAA, IPYA was carried out as previously described (Shindy and Smith, 1975; Guinn et al., 1986). The HPLC parameters were applied as previously described (Tien et al., 1979). The extraction of endogenous ACC was carried out according to Lizada and Yang (1979). Derivatization of ACC was done by adding phenylisothiocyanate (Sigma-Aldrich) and the HPLC chromatograms were produced as described (Lanneluc-Sanson et al., 1986). Eight replicate samples were analyzed for the detection of photosynthetic pigments and endogenous auxins, PA, and ACC. All procedures were carried out on seedlings at 12 wps.

### Statistical Analyses

Treatments were arranged in a RCBD in all experiments. Data from the experiments were repeated with similar results, combined and analyzed. Data of PD were transformed into log_10_ cfu g^–1^ dry soil, and R% were arc-sine transformed before analysis of variance (ANOVA) was carried out. Data were subjected to ANOVA and means were compared using Fisher’s Protected LSD Test (*P* = 0.05).

For multidimensional analyses, a PCA was performed to determine the contribution of PGP to the experimental variance. Results were displayed in a biplot. The PCA was carried out using XLSTAT version 2019.3.2 (Addinsoft, United States). Pearson’s correlation was conducted between all pairs of studied variables within the same treatment of actinobacterial isolates and plant tissues. For all statistical analyses carried out in this study, SAS Software version 9 was used (SAS Institute Inc., NC, United States).

## Results

### Isolation of Halotolerant Actinobacteria From *Salicornia* Rhizosphere Soils

The density of actinobacteria populations in *S. bigelovii* rhizosphere was 5.36 ± SE 1.33 log_10_ cfu g^–1^ dry soil. Thirty-nine actinobacteria were isolated from the rhizosphere on ISSA plates (Supplementary Figure S1). According to their cultural and morphological characteristics, 27 (69.23%) and 12 (30.76%) were identified as SA and NSA isolates, respectively. All *Streptomyces* spp. and genera of the NSA (*Actinomadura, Actinoplanes, Microbispora, Micromonospora*, *Rhodococcus*, *Nocardia*, *Nocardiopsis*, and *Streptosporangium* spp.), were enumerated (Supplementary Figure S1) and purified on OMYEA plates.

Eventhough all 39 isolates grew well and sporulated heavily on ISSA medium supplied with up to 40 g L^–1^ NaCl, only 22 (56.41%) showed increased tolerance to 80 g L^–1^ of NaCl (Supplementary Figure S1). This indicates the efficiency of the selected isolates to tolerate high concentration of NaCl (8%) and were considered to be true halotolerant isolates. Of the 22 halotolerant isolates, 13 (59.1%) and (9) (40.9%) isolates belonged to SA and NSA, respectively.

### Preliminary Screening of Actinobacterial Isolates for Root Colonization of *S. bigelovii*

In order to determine the ability of the halotolerant actinobacterial isolates to colonize *S. bigelovii* roots, an *in vitro* indicator root colonization plate assay was performed. Eight of halotolerant actinobacterial isolates (#1, #3, #10, #14, #23, #29, #33, and #37) failed to colonize the roots of *S. bigelovii* at 8 days after emergence. Therefore, they were excluded from the subsequent studies. The remaining 14 isolates (#2, #4, #6, #7, #9, #11, #15, #18, #21, #25, #27, #31, #32, and #35) showed different degrees of root colonization after 8 days of radicle emergence; 10 isolates (#2, #4, #6, #7, #11, #18, #21, #25, #27, and #32) revealed 100% of root colonization, whilst the rest (#9, #15, #31, and #35) colonized 50–75% of the roots. The latter four isolates were excluded in further studies. Only the 10 isolates showing whole root colonization (7 SA and 3 NSA) were selected.

**FIGURE 1:**
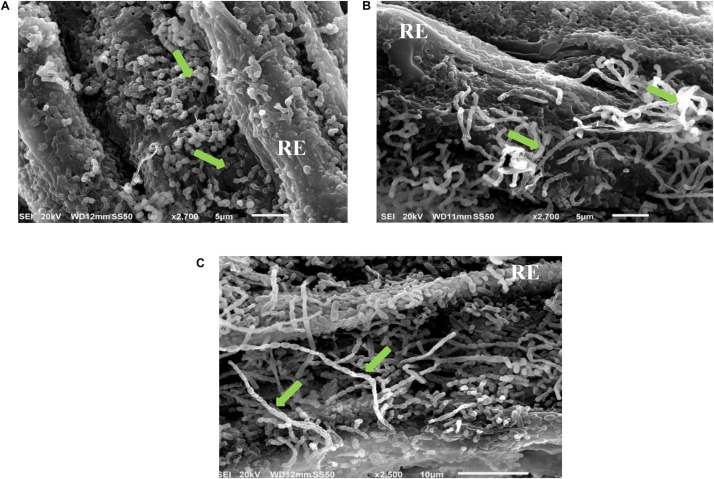
*In vitro* root colonization of *Salicornia bigelovii* by selected halotolerant actinobacterial isolates. Scanning electron micrograph of selected actinobacterial **(A)** auxin-producing isolate #21 (2,700X); **(B)** polyamine-producing isolate #7 (2,700X); and **(C)** ACCD-producing isolate #11 (2,500X) colonizing root of *S. bigelovii*. In **(A–C)**, green arrows represent the chain and/or structure of spores. RE, root epidermis. ACCD, 1-aminocyclopropane-1-carboxylic deaminase.

The full colonization of the 10 isolates for *S. bigelovii* root with plate assay was confirmed by using SEM. For example, it was evident that the actinobacterial isolates #7, #11, and #21 that fully colonized the root system when evaluated by the *in vitro* root colonization plate assay. They also showed extensive mycelial growth and spore chains on the root surface of *S. bigelovii* with the use of SEM (Figure 1).

### Production of IAA, PA, and ACCD by Actinobacteria

Using the colorimetric analysis, auxins were detected in the liquid cultures of isolates #4, #18, #21, #27, and #32 (Table 1). The efficiency of these isolates in their capability for IAA production varied greatly. The other five isolates did not produce IAA. The isolates that formed dark red color after the addition of Salkowski reagent were considered as IAA-producing isolates (Supplementary Figure S2).

**TABLE 1:** Production of indole-3-acetic acid (IAA), polyamines and 1-aminocyclopropane-1-carboxylic acid deaminase (ACCD) by the selected actinobacterial isolates obtained from *Salicornia bigelovii* rhizosphere.

Isolate^a^	IAA equivalents (μg mL^–1^)	Polyamines			ACCD activity (nanomoles α keto-butyrate mg^–1^ protein h^–1^)
		Put (mg L^–1^)	Spd (mg L^–1^)	Spm (mg L^–1^)	
#2	ND	312.13 ± 7.48 *a*	71.75 ± 4.69 *a*	ND	247.78 ± 8.29 *a*
#4	29.76 ± 1.92 *a*	385.50 ± 6.89 *b*	95.87 ± 4.65 *b*	25.79 ± 2.40 *a*	402.98 ± 5.78 *b*
#6	ND	ND	ND	ND	448.14 ± 7.19 *c*
#7^b^	ND	503.59 ± 11.19 *c*	204.70 ± 8.28 *c*	54.46 ± 4.32 *b*	ND
#11^*b*^	ND	ND	ND	ND	440.02 ± 8.32 *c*
#18	20.26 ± 1.21 *b*	ND	ND	ND	ND
#21^b^	2.88 ± 0.87 *c*	ND	ND	ND	ND
#25	ND	432.26 ± 9.78 *c*	135.43 ± 7.18 *d*	57.14 ± 1.68 *b*	ND
#27	10.98 ± 1.04 *d*	ND	ND	ND	118.67 ± 5.22 *d*
#32	17.89 ± 0.75 *e*	278.01 ± 5.98 *d*	68.01 ± 4.89 *a*	38.63 ± 1.27 *c*	ND

*^*a*^#4, #6, #7, #11, #21, #25, and #32 belonged to streptomycete actinobacteria; whilst #2, #18, and #27 belonged to non-streptomycete actinobacteria. ^*b*^#7, #11, and #21 represent Streptomyces tritolerans RAK1, S. rochei RAMS1, and S. chartreusis UAE1, respectively. Values are means of eight independent replicates ± SE for each sampling from two independent experiments. Mean values followed by different letters are significantly (P < 0.05) different from each other according to Fisher’s Protected LSD Test. Put, putrescine; Spd, spermidine; Spm, spermine; ND, non-detectable.*

We also screened the isolates for the ability to produce Put on MDAM plates amended with L-arginine-monohydrochloride. The relatively moderate to large dark red halo around and beneath the colonies in isolates #2, #4, #7, #25, and #32 indicated the production of Put (Table 1 and Supplementary Figure S2): but the absence of red halo was an indicator of inability of the remaining isolates to produce Put. From the HPLC analysis of the culture extracts of the 10 tested isolates grown on MDBM amended with L-arginine-monohydrochloride, the production of the PA (Put, Spd, and Spm) varied significantly (*P* < 0.05) (Table 1).

Five out of the 10 isolates grew and sporulated on DF + ACC agar (Supplementary Figure S2); the rest, however, grew only on DF control medium with (NH_4_)_2_SO_4_ (Table 1). Quantitative estimation of ACCD activity revealed significant (*P* < 0.05) variations in the production of ACCD among the different isolates (Table 1). We noticed that isolates #2, #4, #6, #11, and #27 produced moderate to high levels of ACCD activity and were considered ACCD producers for further analyses.

### Rhizosphere Competence Assays Under Naturally Competitive Environment

The rhizosphere competence assays were carried out to evaluate the root colonization abilities under a naturally competitive greenhouse environment for the 10 isolates that initially colonized the entire root system *in vitro*. There was significant (*P* < 0.05) variation in the root colonizing abilities of the different isolates (Table 2). The root-colonization frequencies of actinobcaterial strains in the rhizosphere soil were highest in isolates #7, #11, #21, and #27 that successfully colonized both the root and the rhizosphere up to 12 cm of root length (Table 2). Although the 10 isolates colonized the rhizosphere soil at all soil depths, their abundancies were significantly (*P* < 0.05) greater at the top 8 cm in comparison to deeper roots (Table 2). All 10 isolates colonized the root system of *S. bigelovii* and the rhizosphere soil (up to 12 cm depth), but isolates #11, #7, #21, and #27 showed the highest colonized PD values.

**TABLE 2:** Root colonization frequencies (%) of the auxins, polyamines and the 1-aminocyclopropane-1-carboxylic acid deaminase producing actinobacterial isolates in root segments (R) of *Salicornia bigelovii* and population densities (PD) (Mean log_10_ cfu g^–1^ dry soil) of the actinobacterial isolates in rhizosphere soil of *S. bigelovii* at 3 weeks post-sowing (wps) treated seeds in the non-sterile soil tube rhizosphere competence assay.

Distance from seed (cm)*^a^*	Isolate																			
	#2		#4		#6		#7*^b^*		#11*^b^*		#18		#21*^b^*		#25		#27		#32	
	R	PD	R	PD	R	PD	R	PD	R	PD	R	PD	R	PD	R	PD	R	PD	R	PD
0–2	100 a	5.02 a	100 a	4.41 a	100 a	3.91 a	100 a	5.55 a	100 a	6.35 a	100 a	3.65 a	100 a	5.33 a	100 a	4.42 a	100 a	4.83 a	100 a	4.11 a
2–4	100 a	4.39 b	100 a	3.65 b	100 a	3.33 b	100 a	5.42 a	100 a	5.72 b	100 a	2.97 b	100 a	4.85 b	90 a	3.72 b	100 a	4.22 b	100 a	4.05 a
4–6	100 a	3.78 c	70 b	3.02 c	100 a	3.21 b	100 a	4.72 b	100 a	5.61 b	100 a	2.45 c	100 a	4.43 c	70 b	3.11 c	100 a	4.05 b	80 b	3.25 b
6–8	100 a	3.15 d	50 c	2.45 d	80 b	2.55 c	100 a	4.65 b	100 a	4.86 c	100 a	2.57 c	100 a	4.31 c	60 b	2.55 d	100 a	3.66 c	65 c	2.66 c
8–10	80 b	2.45 e	40 c	1.56 e	60 c	2.45 c	100 a	4.18 c	100 a	4.74 c	70 b	1.94 d	100 a	3.89 d	40 c	1.82 e	100 a	3.41 c	60 c	2.05 d
10–12	60 c	1.88 f	20 d	1.47 e	30 d	1.58 d	100 a	4.25 c	100 a	4.93 c	50 c	1.25 e	100 a	3.72 d	35 c	1.22 f	100 a	3.55 c	30 d	1.52 e

*^*a*^S. bigelovii seeds were coated with each isolate and grown in non-sterile field sandy soils in plastic tubes in polystyrene boxes containing soil, in an evaporative-cooled greenhouse maintained at 25 ± 2°C. Seedlings were harvested 3 wps the seeds. ^*b*^Isolates #7, #11, and #21 represent Streptomyces tritolerans RAK1, S. rochei RAMS1, and S. chartreusis UAE1, respectively. Values are means of 16 independent replicates for each treatment from two independent experiments. Values with the same letter within a column are not significantly (P > 0.05) different according to Fisher’s Protected LSD Test. Percentage data were arc-sine transformed before analysis.*

The strongest three rhizosphere-competent isolates #21, #7, and #11 that also were good producers for auxins, PA and ACCD, respectively, were selected to study their effects on *S. bigelovii* growth under greenhouse conditions. Notably, isolates #2, #4, #6, #18, #25, and #32 produced high levels of auxins, PA and/or ACCD (Table 1). However, none of the isolates were selected for the greenhouse experiments because they showed low levels of rhizosphere competency (Table 2). Similarly, isolate #27 was eliminated due to its multiple modes of action albeit the excellent rhizosphere competency. In this study, the criteria used in choosing the isolates were (1) to solely possess a single mechanism of action such as producing high levels of auxins, PA or ACCD; and (2) to be able to show a strong rhizosphere competency as recommended by El-Tarabily et al. (2020).

### Identification and Characterization of the Halotolerant Rhizosphere-Competent Isolates

**FIGURE 2:**
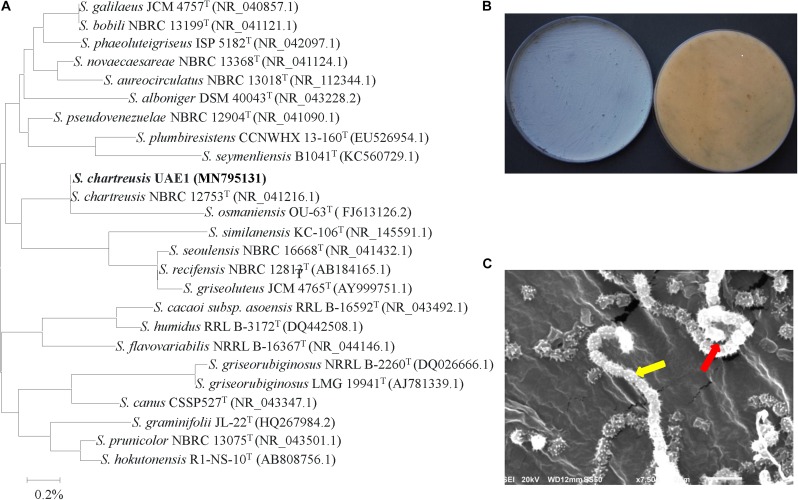
Taxonomic characterization of *Streptomyces chartreusis* UAE1. **(A)** The tree showing the phylogenetic relationships between the auxin-producing *S. chartreusis* UAE1 (isolate #21; MN795131; 1,519 bp) and other members of *Streptomyces* spp. on the basis of 16S rRNA sequences. **(B)** Aerial mycelia (left) and substrate mycelia (right) growing on ISP3 medium supplemented with yeast extract; and **(C)** scanning electron micrograph (7,500X) showing spiral chains of spores (red arrow) with spiny spore structure (yellow arrow) of the strain of *S. chartreusis* UAE1. In **(A)** numbers at nodes indicate percentage levels of bootstrap support based on a neighbor-joining analysis of 1000 resampled datasets. Bar, 0.002 substitutions per site. GenBank accession numbers are given in parentheses.

The promising isolates that only produced either auxins, PA or ACCD isolates were further identified and characterized. Purified PCR products of the 16S rDNA gene was sequenced. The resulting sequence data of isolates #21 (Genbank accession number: MN795131), #7 (MN795132), and #11 (MN795133) were compared with representative 16S rRNA gene sequences of organisms belonging to the actinobacteria. Based on the evolutionary distance values, the phylogenetic tree was constructed according to the neighborhood joining method (Saitou and Nei, 1987). The 16S rRNA gene of isolates #21, #7, and #11 were compared with sequences in the GenBank, European Molecular Biology Laboratory (EMBL) or Ribosomal Database Project (RDP) database (Maidak et al., 1999), which showed that these actinobacterial candidates were all belonged to the genus *Streptomyces* spp. The complete 16S rRNA gene sequence of isolate #21 showed full 100.0% similarity with *S. chartreusis* (NR 041216), although the remaining isolates of *Streptomyces* spp. showed less than 99.3% similarities (Figure 2A). On ISP3 medium, pure cultures of isolate #21 produced powdery blue-green aerial mycelium with yellow substrate mycelial growth after 7 days of incubation (Figure 2B and Supplementary Table S1). According to our observations, the isolate showed branched substrate mycelia from which aerial hyphae developed in the form of open spirals (Figure 2C). The configuration of the spore chains of isolate #21 which belonged to spirales section of 10–25 mature, spiny spores per chain was detected (Figure 2C and Supplementary Table S1). Our data suggest that isolate #21 can be recognized as *Streptomyces chartreusis* Leach et al. (1953) strain UAE1.

**FIGURE 3:**
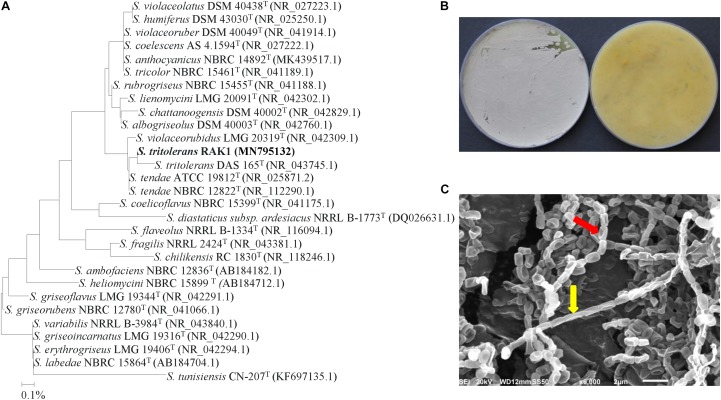
Identification of *Streptomyces tritolerans* RAK1 based on phylogenetic, cultural and morphological characteristics. **(A)** The tree showing the phylogenetic relationships between the polyamine-producing *S. tritolerans* RAK1 (isolate #7; MN795132; 1,516 bp) and other members of *Streptomyces* spp. on the basis of 16S rRNA sequences. **(B)** Aerial mycelia (left) and substrate mycelia (right) growing on ISP3 medium supplemented with yeast extract; and **(C)** scanning electron micrograph (6,000X) showing closed straight to flexuous (rectus-flexibilis) chains of spores (red arrow) with smooth spore structure (yellow arrow) of the strain of *S. tritolerans* RAK1. In **(A)** numbers at nodes indicate percentage levels of bootstrap support based on a neighbor-joining analysis of 1000 resampled datasets. Bar, 0.001 substitutions per site. GenBank accession numbers are given in parentheses.

The phylogenetic analysis of isolate #7 showed 99.6% similarity to *S. tritolerans* DAS 165 (NR 043745) and *S. tendae* ATCC (NR 025871) and NBRC 12822 (NR 112290); while the rest showed < 99.2% similarity with this particular strain (Figure 3A). This **s**uggests that this isolate could be *S. tritolerans*, *S. tendae* or a strain of a new species within the genus *Streptomyces*. The pure cultures produced dark yellow substrate mycelia and gray aerial mycelia when isolate #7 was incubated for 7 days on ISP3 medium (Figure 3B). The isolate did not produce any diffusible pigments in ISP3 medium, and it grew on plates of 7% of NaCl or more (Supplementary Table S2); all of these distinctive characteristics are common features of *S. tritolerans* (Syed et al., 2007). Spores of the isolate were smooth, oval-rod shaped (Figure 3C); and its spore chains arranged in long straight to flexous (rectiflexibiles) chains of 10–15 spores (Figure 3C and Supplementary Table S2). Our data further support the identification of isolate #7 can be recognized as *Streptomyces tritolerans* Syed et al. (2007) strain RAK1.

**FIGURE 4:**
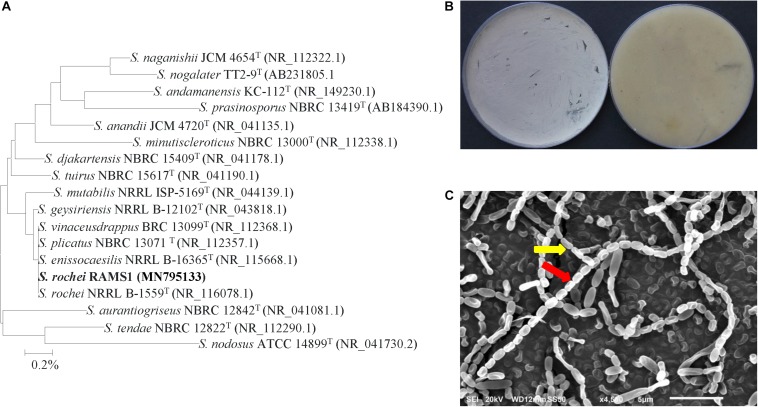
Taxonomic determination of *Streptomyces rochei* RAMS1. **(A)** The tree showing the phylogenetic relationships between the ACCD-producing *S. rochei* RAMS1 (isolate #11; MN795133; 1,519 bp) and other members of *Streptomyces* spp. on the basis of 16S rRNA sequences. **(B)** Aerial mycelia (left) and substrate mycelia (right) growing on ISP3 medium supplemented with yeast extract, and **(C)** scanning electron micrograph (6,000X) showing closed straight to flexuous (rectus-flexibilis) chains of spores (red arrow) with smooth spore structure (yellow arrow) of the strain of *S. rochei* RAMS1. In **(A)** numbers at nodes indicate percentage levels of bootstrap support based on a neighbor-joining analysis of 1000 resampled datasets. Bar, 0.001 substitutions per site. GenBank accession numbers are given in parentheses. ACCD, 1-aminocyclopropane-1-carboxylic deaminase.

For isolate #11, it was phylogenetically grouped in the same clade as five other species, namely *S. geysiriensis* NRRL B-12102 (NR 043818), *S. vinaceusdrappus* BRC 13099 (NR 112368), *S. plicatus* NBRC 13071 (NR 112357), *S. enissocaesilis* NRRL B-16365 (NR 115668), and *S. rochei* NRRL B-1559 (NR 116078) with 100% similarity (Figure 4A). To identify our isolate from the very closely related species, detailed morphological, cultural, biochemical and physiological characterization were carried out to distinguish the ACCD-producing isolate. Light grayish-yellow color of aerial mycelium and pale grayish-yellow color of substrate mycelium were produced by isolate #11 on ISP3 medium without production of any diffusible pigments (Figure 4B). Except of *S. enissocaesilis*, it was difficult to distinguish between our isolate and the other four species based on the aerial mass or substrate mycelium color on any of the ISP media (Supplementary Table S3). At high magnification of SEM, the strain clearly developed smooth, oval-rod shaped spores forming straight to flexuous long chains (rectiflexibiles) of 20–35 spores per chain (Figure 4C). The other *Streptomyces* strains, however, showed either retinaculum-apertum or spiral spore chains (Supplementary Table S3). The identification was further confirmed when other phenotypic, biochemical and physiological characteristics between isolate #21 and *S. rochei* NRRL B-1559 (NR 116078) (Kämpfer, 2012) were comparable (Supplementary Table S3); suggesting that isolate #11 can most probably be *Streptomyces rochei* Berger et al. (1953) strain RAMS1. Together, our data indicate that the auxins, PA, and ACCD-producing isolates were identified as *S. chartreusis*, *S. tritolerans* and *S. rochei*, respectively.

### Assessment of PGP Activities by the Auxin-, PA-, and ACCD-Producing Isolates

The production of the PGRs, ACCD and siderophores, and P-solubilization abilities varied among *S. chartreusis*, *S. tritolerans*, and *S. rochei* (Table 3). Only the auxin-producing isolate *S. chartreusis* produced high levels of IAA and IPYA (44.35 and 9.11 μg mL^–1^, respectively), but did not produce Put, Spd, Spm, or ACCD. Conversely, the PA-producing isolate *S. tritolerans* (#7) only produced high levels of Put, Spd, Spm (Tables 1, 3), but did not produce detectable levels of IAA, IPYA or ACCD (Tables 1, 3). Unlike the other two strains, the ACCD-producing isolate *S. rochei* (#11) produced only ACCD without the production of detectable levels of IAA, IPYA, Put, Spd, or Spm (Tables 1, 3). We did not notice detectable levels of gibberellic acid in the culture extracts of any of the three isolates (Table 3). There was a variation in the production of cytokinins by the three isolates. Isolates #7 and #11 produced 1.12 and 1.32 μg mL^–1^ of iPa, respectively, and isolates #21 and #11 produced 0.95 and 1.52 μg mL^–1^ of iPA, respectively. The two isolates #21 and #7 produced 1.45 and 1.05 μg mL^–1^ of Z, respectively (Table 3).

**TABLE 3:** *In vitro* production of plant growth regulators, 1-aminocyclopropane-1-carboxylic acid deaminase (ACCD), siderophores, nitrogenase enzyme and ammonia and the phosphorus (P) solubilization ability by *Streptomyces chartreusis* UAE1, *S. tritolerans* RAK1, and *S. rochei* RAMS1 isolated from the rhizosphere of *Salicornia bigelovii*.

Activity	*S. chartreusis* (#21)	*S. tritolerans* (#7)	*S. rochei* (#11)
IAA	+	−	−
IPYA	+	−	−
Put	−	+	−
Spd	−	+	−
Spm	−	+	−
GA_3_	−	−	−
iPa	−	+	+
iPA	+	−	+
Z	+	+	−
ACCD	−	−	+
Siderophores	+	−	−
Nitrogenase	−	−	−
Ammonia	−	−	−
P-solubilization	+	+	−
Tolerance to NaCl (8%)	++	++	++

*Data are from eight independent replicates. IAA, indole-3-acetic acid; IPYA, indole-3-pyruvic acid; Put, putrescine; Spd, spermidine; Spm, spermine; GA_3_, gibberellic acid; iPa, isopentenyl adenine; iPA, isopentenyl adenoside; Z, zeatin. +, producing; −, not producing detectable levels; ++, highly tolerant to NaCl (8%).*

The three tested isolates neither fixed N nor produced ammonia. Only two of the isolates (#7 and #21) were able to solubilize P and only one isolate (#21) was able to produce siderophores (Table 3 and Supplementary Figure S2).

Interestingly, the growth of *S. chartreusis*, *S. tritolerans*, and *S. rochei* did not appear to be affected by the presence of each other; there was no evidence of inhibitory effects of the volatile metabolites on the isolates’ growth. In addition, the sprayed target isolates were not inhibited by the spot-inoculated isolate, indicating no observed inhibitory effect of the diffusible metabolites on the growth of the target isolates.

### Assessment of Growth Promotion of *S. bigelovii* Under Greenhouse Conditions

**FIGURE 5:**
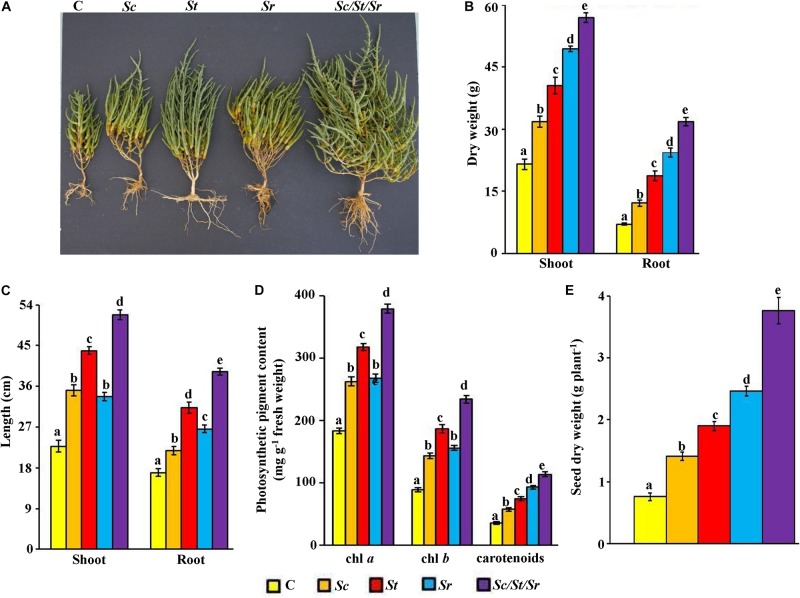
Effect of the rhizosphere-competent actinobacterial isolates on growth and seed yield of *Salicornia bigelovii*. Effect of the treatment of the rhizosphere-competent auxin-, polyamine-, and ACCD-producing isolates on **(A)** performance; **(B)** dry weight and **(C)** length of shoot and root tissues; **(D)** photosynthetic pigment contents of chl *a*, chl *b* and carotenoids*;* and **(E)** dry weight of seeds, of *S. bigelovii*. In **(A–D)**, the figure, dry weight and length of shoot and root tissues, and photosynthetic pigments were measured at 12 wps the seeds; whereas dry weight of seeds was measured at 20 wps. Values are means of 16 replicates for each sampling from two independent experiments. Mean values followed by different letters are significantly (*P* < 0.05) different from each other according to Fisher’s Protected LSD Test. Bars represent standard error. C, control (autoclaved non-inoculated oat bran); *Sc*, auxin-producing isolate #21 (*S. chartreusis* UAE1); *St*, polyamine-producing isolate #7 (*S. tritolerans* RAK1); *Sr*, ACCD-producing isolate #11 (*S. rochei* RAMS1); *Sc*/*St*/*Sr*, consortium of the three rhizosphere-competent isolates. ACCD, 1-aminocyclopropane-1-carboxylic deaminase; chl, chlorophyll; wps, weeks post-sowing.

In order to determine the effect of the three actinobacterial isolates as producers for specific PGP, we treated *S. bigelovii* with each isolate individually and in combinations (i.e., as a synergistic consortium). In general, the application of *S. chartreusis* (*Sc*), *S. tritolerans* (*St*), and *S. rochei* (*Sr*) either singly or in combination (*Sc*/*St*/*Sr*) significantly (*P* < 0.05) promoted growth of *S. bigelovii* compared to the control treatment after 12 wps of sowing *S. bigelovii* seeds (Figure 5A). Compared to the non-inoculated control plants, *Sc* treatment increased shoot and root dry biomass by 32.3 and 42.3%, respectively (Figure 5B). When seawater-irrigated plants were treated with either *St* or *Sr*, there was a significant (*P* < 0.05) increase in the dry biomass of shoots (46.9% in *St* and 56.5% in *Sr*) and roots (62.4% in *St* and 71.9% in *Sr*) compared to the control treatment. Similar observations were found in shoot and root tissues when we measured their lengths. When we treated *S. bigelovii* seedlings with *Sc*, *St* or *Sr*, we found that the length of both shoots and roots increased significantly (*P* < 0.05) compared to control (Figure 5C). Thus, *Sc*/*St*/*Sr* revealed the greatest growth. This was confirmed by the significant increases in the dry weights of shoots and roots by 62.2 and 77.9%, respectively (Figure 5B), and the length of shoots and root by 56.0 and 56.8%, respectively (Figure 5C) compared to non-inoculated control plants.

Treatments of *S. bigelovii* with the three individual isolates (*Sc*, *St*, or *Sr*) or with the combined approach (*Sc*/*St*/*Sr*) had significantly (*P* < 0.05) increased levels of chl *a*, chl *b* and carotenoids as compared with control plants (Figure 5D). The combined effect of the three isolates (*Sc*/*St*/*Sr*) resulted in more production of photosynthetic pigments compared the application of each isolate individually. Similarly, *S. bigelovii* yielded more seeds of 46.1% in *Sc*, 60.0% in *St* and 69.1% in *Sr* than that in the control plants watered with seawater at the harvest time (Figure 5E). Such increase in seed yield reached to 79.7% in the consortium treatment of *Sc*/*St*/*Sr* when compared to the control plants. In individual treatments, growth enhancement reflected as a set of morpho-physiological parameters (dry biomass, photosynthetic pigments and seed yield), was most pronounced with the ACCD-producing *S. rochei* (Figure 5). This was followed by the PA-producing *S. tritolerans* and lastly by the auxin-producing isolate *S. chartreusis* compared to non-inoculated control plants. Interestingly, the consortium had the superiority in growth promotion effect in comparison with any other individual applications. This suggests that the consortium of actinobacterial isolates stimulated growth of *S. bigelovii*, most probably, due to the synergistic interactions between these isolates via multiple mechanisms.

### Determination of the Levels of Auxins, PA, and ACC *in planta*

**FIGURE 6:**
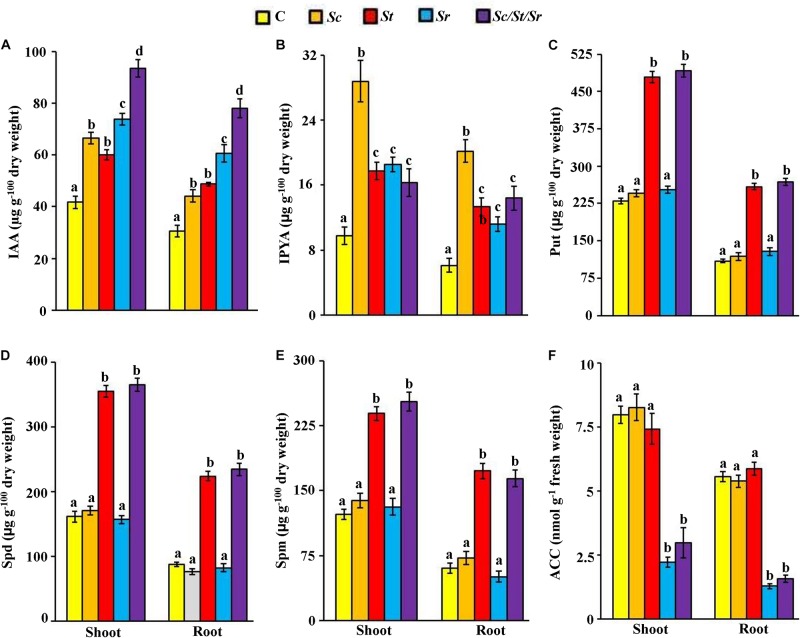
Effect of the individual and/or consortium of the rhizosphere-competent actinobacteria on endogenous auxins, polyamines and ACC content of *Salicornia bigelovii.* Endogenous contents of **(A)** IAA; **(B)** IPYA; **(C)** Put; **(D)** Spd; **(E)** Spm, and **(F)** ACC in *S. bigelovii* shoot and root tissues after treatment with autoclaved non-inoculated oat bran (control); auxin-producing isolate #21 (*S. chartreusis* UAE1, *Sc*); polyamine-producing isolate #7 (*S. tritolerans* RAK1, *St*); ACCD-producing isolate #11 (*S. rochei* RAMS1, *Sr*); consortium of the three rhizosphere-competent isolates (*Sc*/*St*/*Sr*). *S. bigelovii* seedlings were grown in an evaporative-cooled greenhouse and maintained at 25 ± 2°C. Values are means of eight replicates for each sampling from two independent experiments. Mean values followed by different letters are significantly (*P* < 0.05) different from each other according to Fisher’s Protected LSD Test. Bars represent standard error. Endogenous contents of IAA, IPYA, Put, Spd, Spm, and ACC were measured at 12 wps the seeds of *S. bigelovii*. IAA, indole-3-acetic acid; IPYA, indole-3-pyruvic acid; Put, putrescine; Spd, spermidine, Spm, spermine; ACC, 1-aminocyclopropane-1-carboxylic; ACCD, ACC deaminase; wps, weeks post-sowing.

To study possible mechanisms leading to the growth enhancement of *S. bigelovii* after individual or combined treatments of isolates, we analyzed the endogenous levels of particular PGRs in shoot and root tissues of *S. bigelovii*. The application of *Sc* or *Sc*/*St*/*Sr* significantly (*P* < 0.05) increased the levels of IAA and IPYA in the tissues of *S. bigelovii* compared to control plants (Figures 6A,B). Plants treated with either the *St* or the mixture of isolates significantly (*P* < 0.05) increased the levels of endogenous Put, Spd and Spm than any other treatment including the control in both roots and shoot tissues (Figures 6C–E). Similarly, *S. bigelovii* treated with *Sr* or *Sc*/*St*/*Sr* significantly (*P* < 0.05) reduced the levels of ACC in the plant tissues compared to the control treatment (Figure 6F).

Application with the PA-producing isolate *S. tritolerans* (*St*) or the ACCD-producing *S. rochei* (*Sr*) significantly (*P* < 0.05) increased levels of endogenous auxins of *S. bigelovii* compared to control (Figures 6A,B). Both *S. tritolerans* and *S. rochei* were, however, found to be non-auxin-producing isolates. This suggests that the production of PA and ACCD by these isolates may indirectly increase the auxins levels *in planta*.

In general, the overall endogenous levels of auxins and PA measured in both tissues of *S. bigelovii* were found to be high, but low levels of ACC in *Sc*/*St*/*Sr* treatment were detected compared with other treatments (Figure 6). This suggests that the synergistic consortium of isolates positively regulated the activities of the endogenous hormone auxin and the PA with a concomitant decline in the level of ACC *in planta* more than those in the individual treatments. This ultimately could lead to better growth and performance of *S. bigelovii*. To lesser extent, the effects were also observed by the individual application of *Sc*, *St* or *Sr*.

### Assessment of Plant Growth Promotion Using Multivariate Analysis

**FIGURE 7:**
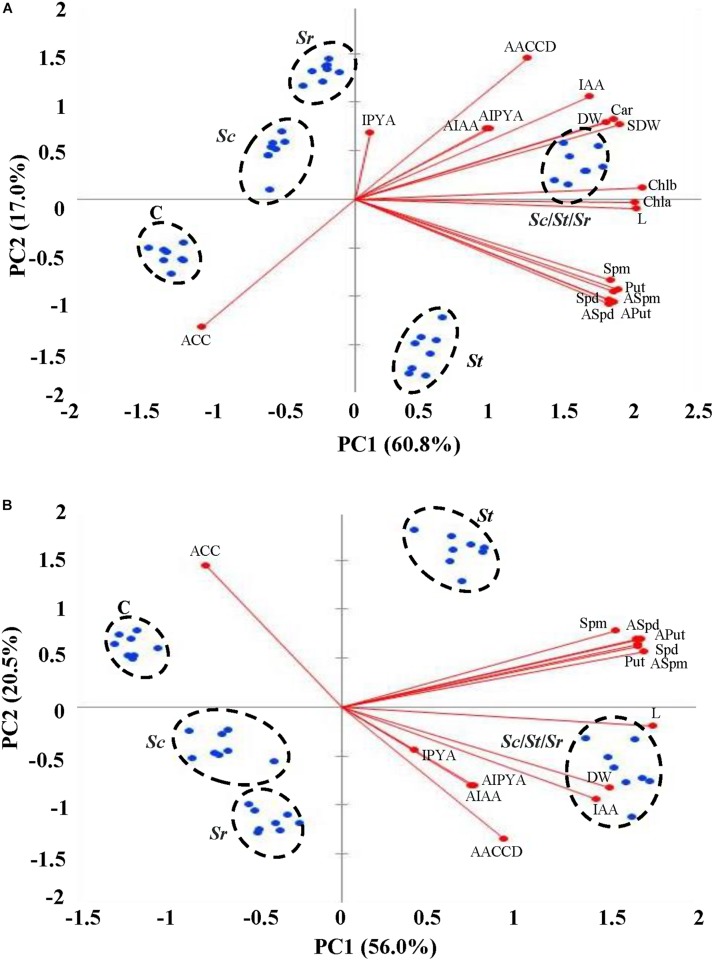
Multivariate statistical analyses of the endogenous PGRs of *Salicornia bigelovii* treated with actinobacterial isolates. Principal component (PC) analysis on **(A)** shoot and **(B)** root tissues of *S. bigelovii* after treatment with autoclaved non-inoculated oat bran (control); auxin-producing isolate #21 (*S. chartreusis* UAE1, *Sc*); PA-producing isolate #7 (*S. tritolerans* RAK1, *St*); ACCD-producing isolate #11 (*S. rochei* RAMS1, *Sr*) consortium of the three rhizosphere-competent isolates (*Sc*/*St*/*Sr*). Two auxins (AIAA and AIPYA), three PA (Aput, ASpd, and Aspm) and (AACCD) obtained from actinobacterial isolates, eight agronomic growth parameters (L and DW in shoot and root separately, chl *a*, chl *b*, and Car in shoot, and SDW) and six plant growth regulators (IAA, IPYA, Put, Spd, Spm, and ACC) obtained from *S. bigelovii*, were tested. *S. bigelovii* seedlings were grown in an evaporative-cooled greenhouse and maintained at 25 ± 2°C. Values are means of eight replicates for each sampling from two independent experiments. IAA, indole-3-acetic acid; IPYA, indole-3-pyruvic acid; Put, putrescine; Spd, spermidine, Spm, spermine; ACC, 1-aminocyclopropane-1-carboxylic; PA, polyamines; ACCD, ACC deaminase; L, length; DW, dry weight; Chl, chlorophyll; Car, carotenoids; SDW, seed dry weight.

The PCA was performed to identify the principal components (PCs) to identify the most important morpho-physiological parameters that are responsible about the variation of both shoot and root growth and seed yield of *S. bigelovii* that best describe the growth promotion to the actinobcaterial isolates individually or in combination. The first two PCs accounted for 60.8 and 17% of the total variation in shoots (77.8%; Figure 7A) and 56 and 20.5% of the total variation in roots (76.5%; Figure 7B). Plants received the five different treatments were segregated into clear five clusters; plants that were not treated with any actinobacterial isolate (control) were on side, but plants inoculated with the combination of the three actinobcaterial isolates (*Sc/St/Sr)* on the other side that had higher agronomic growth parameters associated positively with all PGRs in plant tissues. Plants treated with the auxin-producing *S. chartreusis* are located next to control plants followed by plants treated with ACCD-producing *S. rochei*. The relatively slower growth and seed yield of *Sc* and *Sr* treated plants, as compared to those treated with *St* and *Sc/St/Sr* were not associated with insignificant productions of Put, Spd, Spm and ACC. In fact, ACC was negatively associated in roots of plants treated with *Sc* and both roots and shoots of plants treated with *Sr*. The Scree plots representing the eigenvalue of variance explained by each PC in shoot and root were also shown (Supplementary Figure S3).

The effects of auxins, PA and/or ACCD produced by individual or combined isolates were also evaluated on plant growth parameters in shoot and root tissues. On the biplots, *Sc*, *St*, and *Sr* were closely located to auxins, PA, and ACCD, respectively (Figure 7). The consortium of isolates, on the other hand, was closely related to all growth parameters and PGRs. In general, the accumulation of growth parameters was positively correlated upon individual or mixed inoculations of isolates, but was negatively correlated with the endogenous ACC content of shoot and root. This suggests that each strain of actinobacteria possessing different mechanisms can directly contribute to plant promotion; thus the application of the consortium showing rhizopshere-competent abilities maximizes the growth and yield of *S. bigelovii*.

We also studied the association across the morpho-physiological and biochemical characteristics of *S. bigelovii* and the PGP produced by each isolate. The correlation coefficients (*r*-values) analysis showed that the auxin-producing *S. chartreusis* had significant (*P* < 0.05) correlation with all agronomic growth parameters, IAA and IPYA; but insignificant (*P* > 0.05) with the endogenous PA and ACC in both plant organs (Supplementary Tables S4, S5). Thus, 45 and 28 insignificant (*P* > 0.05) correlations with *Sc* in shoot and root, respectively. There were 14 and 10 insignificant (*P* > 0.05) correlations in shoots and roots of *S. bigelovii* inoculated with the PA-producing *S. tritolerans*, respectively (Supplementary Tables S6, S7); and 24 and 23 insignificant correlations in the same tissues with the ACCD-producing *S. rochei* treatment (Supplementary Tables S8, S9). Neither *Sc* nor *St* significantly correlated with ACC. *Sr* negatively correlated with ACC *in planta*. It is noteworthy to mention that most endogenous PA in shoot and root tissues were insignificant (*P* > 0.05) in *S. bigelovii* tissues after *Sr* treatment. This was confirmed when the three isolates were combined to determine the correlation coefficients between plant growth attributes in shoots and roots of *S. bigelovii* and endogenous growth regulation compounds. The synergistic consortium significantly (*P* < 0.01) correlated with almost all agronomic growth parameters and PGRs in plant tissues; and thus the insignificance was not observed between growth regulation parameters in root (Supplementary Tables S10, S11). This indicates that the promoting effects of the ACCD, three PA followed by the auxins detected in *S. rochei*, *S. tritolerans*, and *S. chartreusis*, respectively, were effective in enhancing different levels of growth regulating compounds. Thus, the relative superiority in performance of the consortium can be attributed to the multiple modes of action that are known to enhance growth and relieve plants from environmental stresses.

## Discussion

In the UAE, the production of aviation biofuel can be derived from halophytic plants such as *Salicornia* spp. that can successfully grow in salt marshes. However, the growth and seed yield of plants, even halophytes, under salt conditions are very low. It is known that PGPR can improve plant growth by enhancing nutrient recycling and producing PGRs; thus minimizing the application of chemical fertilization (Hassan et al., 2019). Therefore, PGPR can play an essential role in the sustainable agriculture industry. Specifically, actinobacteria can be more suitable for dry, arid soils and extreme hot environments (Chaurasia et al., 2018) such as those in the UAE (Saeed et al., 2017; Kamil et al., 2018). A recent study has compared growth promotion of *S. bigelovii* between rhizosphere-competent versus rhizosphere-non-competent actinobacterial strains after irrigation with seawater (El-Tarabily et al., 2020). Although both isolates produced high levels of PA, PGP has been more remarkable in the presence of the rhizosphere-competent, *Streptomyces euryhalinus*, than the rhizosphere-non-competent, *Actinoplanes deccanensis*. The outcomes imply the importance of rhizosphere competency as a criterion for PGPR selection. Here, we hypothesized that inoculation with a “consortium” of rhizosphere-competent actinobacteria possessing different PGP activities can consequently improve the growth characteristics of *S. bigelovii* irrigated with seawater.

In this report, the performance of the three halotolerant marine rhizosphere-competent PGP actinobacterial isolates was assessed on seawater-irrigated *S. bigelovii* seedlings. *S. chartreusis* UAE1 (#21), *S. tritolerans* RAK1 (#7), and *S. rochei* RAMS1 (#11) that were isolated from *S. bigelovii* rhizosphere stimulated the growth of seedlings of this halophytic cash crop under greenhouse conditions. Moreover, the application of the mixture treatment consisting of the three isolates was more effective in promoting growth of *S. bigelovii*, indicating synergistic effects rather than individual effects. By using single actinobacterial strains, growth promotion -reflected growth characteristics in tissues of *S. bigelovii*- was most pronounced with *S. rochei* that produced high levels of ACCD. Although seawater-irrigated *S. bigelovii* showed increased length in shoots and roots when treated with the auxin-producing isolate *S. chartreusis*, seedlings treated with the PA-producing isolate *S. tritolerans* produced more biomass and seed yield than those treated with *S. chartreusis*. Thus, the relative superiority in performance of the ACCD*-*producing isolate *S. rochei* and the PA-producing isolate *S. tritolerans* over the auxin-producing isolate *S. chartreusis* might be attributed to their abilities to produce ACCD and PA, respectively, which have been known to relieve plants from environmental stresses (Glick et al., 2007; Ghosh et al., 2018). Contributions of these components in growth enhancement are significant for the ability of PGPR to increase plant tolerance to salinity and facilitate plant growth. Although *S. chartreusis* was effective to a lesser extent than *S. tritolerans* or *S. rochei*, this auxin-producing isolate was still able to significantly improve seedling growth compared with the control treatment. This study highlights, for the first time, the potential of using a mixture of marine rhizosphere-competent actinobacteria as biological inoculants to enhance growth and establishment of *Salicornia* in nutrient impoverished marine soils occurring in coastal environments.

*Salicornia* thrive in environments that are N or P deficient. Previous work on PGPR from *Salicornia* rhizosphere and their effects on *Salicornia* growth has dealt predominantly with N-fixing bacteria (Ozawa et al., 2007; Hrynkiewicz et al., 2019) or P-solubilizing bacteria (Bashan et al., 2000). Except of one study done by Jha et al. (2012), no reports have considered if PGPR capable of fixing-N or solubilizing-P can still promote *Salicornia* growth through the production of ACCD or other PGRs. The bacteria isolated from *Salicornia* roots which can fix N, and produce IAA and ACCD have only been tested on *Salicornia* seed germination and seedling performance under axenic conditions in petri dishes using various NaCl concentrations (Jha et al., 2012). Under gnotobiotic and greenhouse conditions, the endophytic ACCD-producing actinobacterium, *M. chalcea*, and the two PA-producing actinobacteria, *S. euryhalinus* and *A. deccanensis*, enhanced seedling growth when irrigated with full strength seawater (El-Tarabily et al., 2019, 2020). In the current study, the three isolates were selected through a series of tests, which included screening for salinity tolerance, rhizosphere-competency under naturally competitive environment and PGP traits.

It is well-known that effective PGP, PGPR strains must colonize the roots and the introduced inoculum must establish and grow in an ecological habitat that includes indigenous rhizosphere microbial community (Hassan et al., 2019; Rilling et al., 2019). In our study, the potential PGPR isolates were initially selected based on their ability to *in vitro* colonize *S. bigelovii* roots; followed by a rhizosphere competence screening in the greenhouse under naturally competitive environment. Halophilic/halotolerant marine non-actinobacterial isolates colonizing *Salicornia* roots under *in vitro* conditions have previously been reported (Mapelli et al., 2013). Non-actinobacterial isolates from native sediments have also been tested for their abilities to colonize mangrove roots *in vitro* and under non-competitive environments (Bashan et al., 1998; Rojas et al., 2001). On the other hand, the abilities of the halotolerant actinobacteria reported in the current study colonizing the rhizosphere of *Salicornia* and showing effective levels of rhizosphere-competency have rarely been screened under naturally competitive environments. Only one study exists in which rhizosphere-competent PA-producing isolates have been used to promote *S. bigelovii* growth under naturally competitive environment (El-Tarabily et al., 2020). Additional emphasis on rhizosphere-competency was deliberately set to the niche around the root zone critical for the physiological activities of roots in the soil. It was not surprising that isolates producing PGRs and possessing high rhizosphere competency were effective in helping the host plant overcome root growth inhibition in the inhospitable environment. We argue that the success of the rhizosphere-competent PGP actinobacterial isolates was most likely attributed to their abilities to produce relatively high levels of PGRs and/or the activity of ACCD.

In order to be considered as an effective root-colonizing isolate, PGPR should have the ability to colonize the entire root system (Hassan et al., 2019; Rilling et al., 2019). There were 10 isolates that exhibited 100% of root colonization in the preliminary plate assay. Under naturally competitive saline conditions, many isolates were strongly deliberated as rhizosphere-competent; despite others showed no or inadequate levels of competency (Table 2). This confirms the importance of testing the isolates in competitive environment. We noticed that all isolates recovered from the rhizosphere on the whole root with different levels of frequency; thus the PD were highly detected in the top 8 cm of the root system. Notably, *S. chartreusis*, *S. tritolerans*, and *S. rochei* reached as deep as to 12 cm colonizing *S. bigelovii* root system. This indicates that these isolates were favored to be excellent rhizosphere-competent and prominently suitable for seed and soil inoculations.

The three outstanding rhizosphere-competent isolates were chosen based on their exceptional abilities to produce auxins, PA and ACCD *in vitro*. Thus, these isolates were neither N-fixers nor ammonia-producers. In addition, they did not produce any detectable levels of GA_3_. Several marine bacteria have been reported to produce auxins (Jha et al., 2012; Mapelli et al., 2013), cytokinins (Maruyama et al., 1986), PA (Suzuki et al., 1990), GA_3_, or ACCD (Sgroy et al., 2009; Mapelli et al., 2013). Some actinobacteria from the coast of the Arabian Gulf have also been reported to produce PGRs in chemically-defined medium (El-Tarabily et al., 2019, 2020).

In the greenhouse trials, inoculations with the auxin-producing *S. chartreusis* resulted in a significant increase in the levels of endogenous IAA and IPYA in *S. bigelovii* (Figure 6). Consistent with that, Fallik et al. (1989) have reported increases in the level of auxins in corn following the application of IAA-producing rhizosphere bacteria. *S. tritolerans* produced considerable amount of the three PA (Put, Spd, and Spm) also stimulated growth of *S. bigelovii*. This was evident when there were significant increases in endogenous PA in the tissues of *S. bigelovii* inoculated with *S. tritolerans* in comparison to plants without inoculation. Similarly, the non-marine PA-producing *Streptomyces griseoluteus* enhanced the growth of bean plants under greenhouse conditions by increasing the endogenous levels of PA (Nassar et al., 2003). Some studies have reported that marine and non-marine bacterial isolates produce PA only (Cassán et al., 2009; Zhou et al., 2016). The third isolate, *S. rochei* with elevated ACCD activity caused significant reduction in the levels of endogenous ACC in roots and shoots; most likely through the cleavage of ACC (the ET precursor) to NH_3_ and α-ketobutyrate (Handa et al., 2010). The growth enhancement in *S. bigelovii* treated with *S. rochei* is supported by other observations of ACCD-producing (actino)bacteria that have also shown to promote plant growth by lowering ET levels (Glick et al., 2007; El-Tarabily, 2008; El-Tarabily et al., 2019). It is known that ET is a major player in plant responses to biotic and abiotic stresses (Mengiste et al., 2010; Sham et al., 2019). As a result, preventing the synthesis of the stress hormone ET to levels inhibitory for the root growth by *S. rochei* could help plant to grow normally as described by Glick et al. (2007). Thus, the activity of ACCD alone was insufficient to reach to the degrees of growth enhancement achieved in the presence of the three isolates together.

Previous studies have reported the advantages of the use of mixed versus individual cultures of PGPR for better growth in mangrove (for review, see Bashan et al., 2004). In *Salicornia* spp., growth promotion and salinity tolerance in the combined treatment were more obvious than in the individual treatment of the rhizosphere or the endophytic bacterial strains (Komaresoflaa et al., 2019). Consistent with that, the application of the actinobacterial consortium increased seed production by 79.7% compared to the control treatment. Mangrove seedlings treated with mixed cultures of *Phyllobacterium* sp. and *Bacillus licheniformis* developed more leaves than by the individual cultures of bacteria (Rojas et al., 2001). The actinobacterial isolates in our study mostly achieved the growth promotion through their ability to solely produce auxins, PA, ACCD and may be certain growth promoting factors other than those assayed on and/or adjacent to the root system. It may also be of interest for future work to study combinations of other isolates belonging to different rhizosphere competency to determine the ranking of importance of various growth factors evaluated.

This is the first study to report *S. chartreusis*, *S. tritolerans*, and *S. rochei* being isolated from *Salicornia* rhizosphere. The PCA and correlation analysis demonstrated the reproducibility of the measurements performed and allowed us to identify possible traits related to treatments of individual or combined inoculation on *S. bigelovii*. We believe this study was successful in obtaining suitable bio-inoculants of *S. bigelovii* seedlings in the UAE or elsewhere. The current study draws the public attention to the feasibility of using halotolerant actinobacteria to promote growth of *S. bigelovii* cultivated in the greenhouse, particularly as a consortium that can potentially be applied in marine farming agricultural systems. Thus, this is expected to increase the large-scale production of *Salicornia* seeds under the recently termed integrated Seawater Energy and Agriculture System (iSEAS)^2^ in order to promote the supply of sustainable aviation biofuels.

## Data Availability Statement

The datasets generated for this study can be found in the Genbank accession number MN795131, MN795132, MN795133.

## Author Contributions

SA and KE-T designed the research, supervised the study, analyzed the data and wrote the manuscript. BM and YT performed greenhouse experiments. BM and KE-T performed the microscopic experiments. BM, AA, A-HM, MA, AE-K, and AH-A assisted with experiments and data evaluation. All authors critically revised the manuscript and approved the final version.

## Conflict of Interest

The authors declare that the research was conducted in the absence of any commercial or financial relationships that could be construed as a potential conflict of interest.

## Footnotes

1 https://blast.ncbi.nlm.nih.gov/Blast.cgi

2 www.ku.ac.ae
