# Supplementary material for: Halotolerant Marine Rhizosphere-Competent Actinobacteria Promote Salicornia bigelovii Growth and Seed Production Using Seawater Irrigation
Source: Front Microbiol. 2020 Apr 3;11:552. doi: 10.3389/fmicb.2020.00552 (PMC7145952; doi:10.3389/fmicb.2020.00552)
Supplement: Supplementary file 1 [file Data_Sheet_1.pdf]

Supplementary Data

Halotolerant marine rhizosphere-competent actinobacteria promote *Salicornia bigelovii* growth and seed production using seawater irrigation

Betty T. Mathew, Yaser Torky, Amr Amin, Abdel-Hamid I. Mourad, Mutamed M. Ayyash, Ali El-Keblawy, Ali Hilal-Alnaqbi, Synan F. AbuQamar* and Khaled A. El-Tarabily*

*** Correspondence:**
Prof. Synan AbuQamar: sabuqamar@uaeu.ac.ae
Prof. Khaled El-Tarabily: ktarabily@uaeu.ac.ae

Supplementary Figures

Figure S1. *In vitro* growth of selected actinobacteria isolated from *Salicornia bigelovii* rhizosphere. **(A)** Colonies of SA and NSA isolated from *S. bigelovii* grown on inorganic salt starch agar plates; and **(B)** selected actinobacterial isolates possessing salinity tolerance. In (A), black arrows represent the SA colonies; whereas red, yellow and green arrows represent the NSA colonies of *Micromonospora*, *Actinoplanes* and *Nocardia* spp., respectively. In **(B)**, actinobacteria #21, #7 and #11, but not #16 (control), were considered halotolerant isolates due to growth and sporulation on inorganic salt starch agar supplemented with 8% NaCl. SA, streptomycete actinobacteria; NSA, non-streptomycete actinobacteria.

Figure S2. *In vitro* plant growth promoting characteristics of selected halotolerant actinobacterial isolates. Selected actinobacterial strains isolated from *Salicornia bigelovii* rhizosphere possessing the production of **(A)** IAA; **(B)** Put; **(C)** ACCD and **(E)** siderophores; and **(D)** solubilization of P. In **(A)**, addition of Salkowski reagent to cultures grown in inorganic salt starch broth amended with L-tryptophan; and the formation of red color indicated the production of the auxin, IAA. In **(B)** isolates were tested on Moeller's decarboxylase agar medium amended with L-arginine-monohydrochloride; and the change from yellow to red color of the phenol-red indicated production of the polyamine, Put. In **(C)** isolates were tested in N-free Dworkin and Foster's salts minimal agar medium amended with ACC; where growth and sporulation indicated the efficiency to utilize ACC and the production of ACCD. In **(D)**, isolates were tested on Pikovskaya's agar medium amended with rock phosphate and bromophenol blue; and production of clear zone surrounding the colony was used as an indicator of P-solubilization. In **(E)**, isolates were tested on chrome azurol S agar plates; and yellow halo surrounding the colony indicates the excretion of siderophores. In **(A-E)**, isolates #21, #7 and #11 were considered as auxin-, polyamine and ACCD-producing isolates, respectively. In **(D & E)**, isolates #21 and #7 were considered as P-solubilizing isolates, whereas #21 was considered as a siderophore-producing isolate. Actinobacterial strain #32 was used as a control isolate. IAA, indole-3-acetic acid; Put, putrescine; ACC, 1-aminocyclopropane-1-carboxylic; ACCD, 1-aminocyclopropane-1-carboxylic deaminase; P, phosphorus.

Table S1. Comparison of morphological, cultural and phenotypic characteristics that distinguish auxin-producing isolate #21 from very closely related species *Streptomyces chartreusis.*

Table S2. Comparison of morphological, cultural and phenotypic characteristics that distinguish polyamine-producing isolate #7 from very closely related species *Streptomyces tritolerans* and *S. tendae.*

Table S3. Comparison of morphological, cultural and phenotypic characteristics that distinguish 1-aminocyclopropane-1-carboxylic deaminase-producing isolate #11 from very closely related species *Streptomyces rochei, S. plicatus, S. enissocaesilis,* and *S. vinaceusdrappus.*

Figure S3. Scree plots representing the eigenvalue of variance accounted for each principal component in **(A)** shoot and **(B)** root. *S. bigelovii* seedlings were grown in an evaporative-cooled greenhouse and maintained at 25±2°C.

Table S4. Pearson correlation coefficient (*n*=8) of 14 variables, including two actinobacterial auxins, six agronomic traits and six plant growth regulators obtained from shoots of *S. bigelovii*. Seedlings were seed- and soil-inoculated with *Streptomyces chartreusis* (*Sc*) grown in an evaporative-cooled greenhouse and maintained at 25±2°C (*, $P<0.05$, **, $P<0.01$ and ***, $P<0.001$).

Table S5. Pearson correlation coefficient (*n*=8) of 10 variables, including two actinobacterial auxins, two agronomic traits and six plant growth regulators obtained from roots of *S. bigelovii*. Seedlings were seed- and soil-inoculated with *Streptomyces chartreusis* (*Sc*) grown in an evaporative-cooled greenhouse and maintained at 25±2°C (*, $P<0.05$, **, $P<0.01$ and ***, $P<0.001$).

Table S6. Pearson correlation coefficient (*n*=8) of 15 variables, including three actinobacterial polyamines, six agronomic traits and six plant growth regulators obtained from shoots of *S. bigelovii*. Seedlings were seed- and soil-inoculated with *Streptomyces tritolerans* (*St*) grown in an evaporative-cooled greenhouse and maintained at 25±2°C (*, $P<0.05$, **, $P<0.01$ and ***, $P<0.001$).

Table S7. Pearson correlation coefficient (*n*=8) of 11 variables, including three actinobacterial polyamines, two agronomic traits and six plant growth regulators obtained from roots of *S. bigelovii*. Seedlings were seed- and soil-inoculated with *Streptomyces tritolerans* (*St*) grown in an evaporative-cooled greenhouse and maintained at 25±2°C (*, $P<0.05$, **, $P<0.01$ and ***, $P<0.001$).

Table S8. Pearson correlation coefficient (*n*=8) of 13 variables, including the actinobacterial 1-aminocyclopropane-1-carboxylic deaminase, six agronomic traits and six plant growth regulators obtained from shoots of *S. bigelovii*. Seedlings were seed- and soil-inoculated with *Streptomyces rochei* (*Sr*) grown in an evaporative-cooled greenhouse and maintained at 25±2°C (*, $P<0.05$, **, $P<0.01$ and ***, $P<0.001$).

Table S9. Pearson correlation coefficient (*n*=8) of nine variables, including the actinobacterial 1-aminocyclopropane-1-carboxylic deaminase, two agronomic traits and six plant growth regulators obtained from roots of *S. bigelovii*. Seedlings were seed- and soil-inoculated with *Streptomyces rochei* (*Sr*) grown in an evaporative-cooled greenhouse and maintained at 25±2°C (*, $P<0.05$, **, $P<0.01$ and ***, $P<0.001$).

Table S10. Pearson correlation coefficient (*n*=8) of 18 variables, including actinobacterial auxins, polyamines and 1-aminocyclopropane-1-carboxylic deaminase, six agronomic traits and six plant growth regulators obtained from shoots of *S. bigelovii*. Seedlings were seed- and soil-inoculated with *S. chartreusis*, *S. tritolerans* and *S. rochei* (*Sc*/*St*/*Sr*) grown in an evaporative-cooled greenhouse and maintained at 25±2°C (*, $P<0.05$, **, $P<0.01$ and ***, $P<0.001$).

Table S11. Pearson correlation coefficient (*n*=8) of 14 variables, including actinobacterial auxins, polyamines and 1-aminocyclopropane-1-carboxylic deaminase, two agronomic traits and six plant growth regulators obtained from roots of *S. bigelovii*. Seedlings were seed- and soil-inoculated with *S. chartreusis*, *S. tritolerans* and *S. rochei* (*Sc*/*St*/*Sr*) grown in an evaporative-cooled greenhouse and maintained at 25 ± 2°C (*, $P<0.05$, **, $P<0.01$ and ***, $P<0.001$).

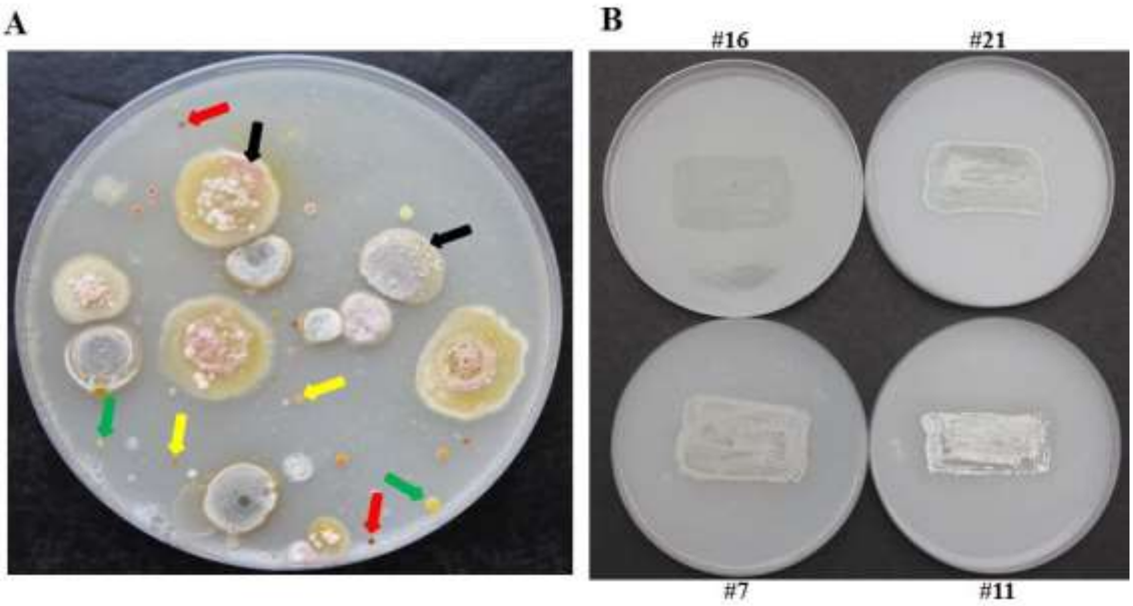

Figure S1. *In vitro* growth of selected actinobacteria isolated from *Salicornia bigelovii* rhizosphere. **(A)** Colonies of SA and NSA isolated from *S. bigelovii* grown on inorganic salt starch agar plates; and **(B)** selected actinobacterial isolates possessing salinity tolerance. In (A), black arrows represent the SA colonies; whereas red, yellow and green arrows represent the NSA colonies of *Micromonospora*, *Actinoplanes* and *Nocardia* spp., respectively. In **(B)**, actinobacteria #21, #7 and #11, but not #16 (control), were considered halotolerant isolates due to growth and sporulation on inorganic salt starch agar supplemented with 8% NaCl. SA, streptomycete actinobacteria; NSA, non-streptomycete actinobacteria.

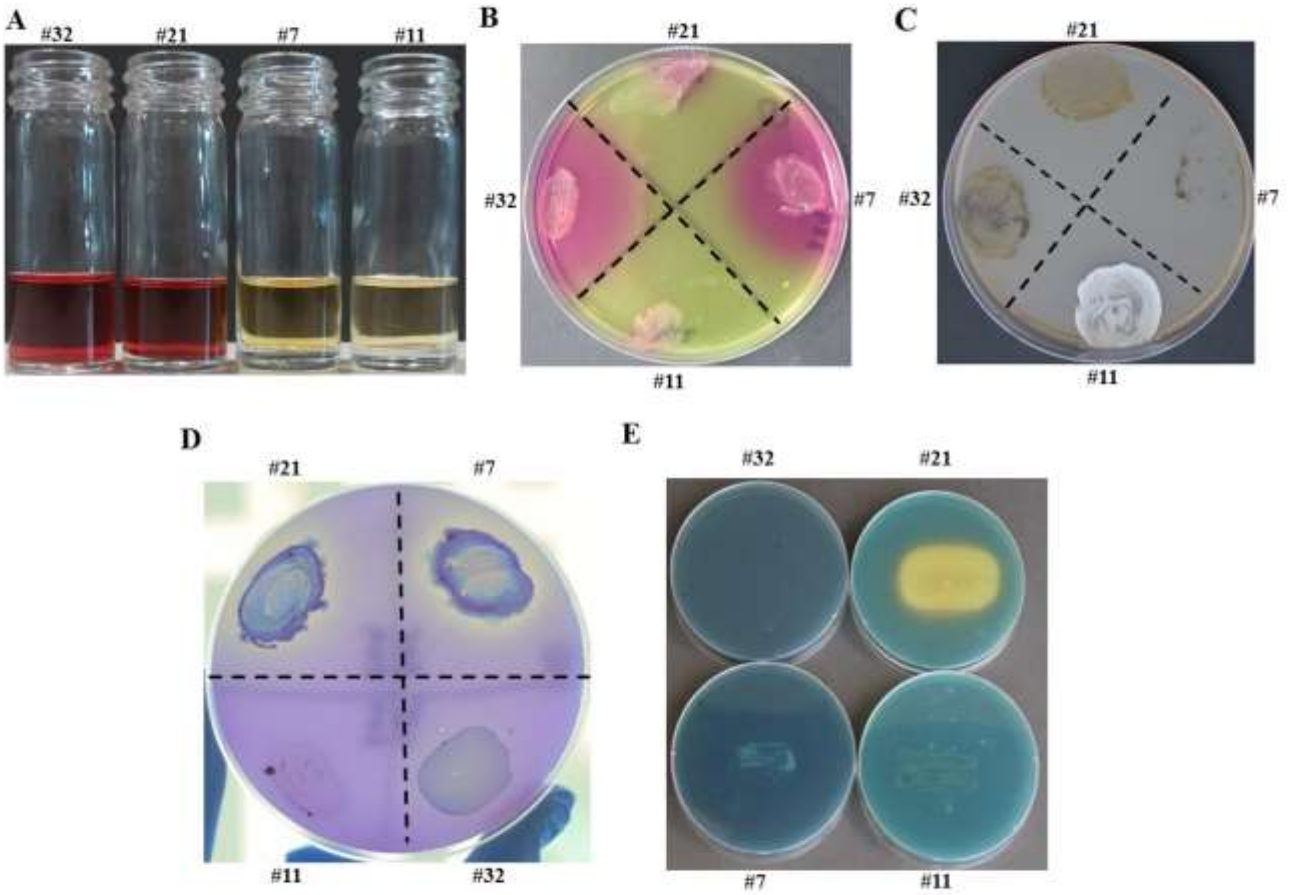

Figure S2. *In vitro* plant growth promoting characteristics of selected halotolerant actinobacterial isolates. Selected actinobacterial strains isolated from *Salicornia bigelovii* rhizosphere possessing the production of **(A)** IAA; **(B)** Put; **(C)** ACCD and **(E)** siderophores; and **(D)** solubilization of P. In **(A)**, addition of Salkowski reagent to cultures grown in inorganic salt starch broth amended with L-tryptophan; and the formation of red color indicated the production of the auxin, IAA. In **(B)** isolates were tested on Moeller's decarboxylase agar medium amended with L-arginine-monohydrochloride; and the change from yellow to red color of the phenol-red indicated production of the polyamine, Put. In **(C)** isolates were tested in N-free Dworkin and Foster's salts minimal agar medium amended with ACC; where growth and sporulation indicated the efficiency to utilize ACC and the production of ACCD. In **(D)**, isolates were tested on Pikovskaya's agar medium amended with rock phosphate and bromophenol blue; and production of clear zone surrounding the colony was used as an indicator of P-solubilization. In **(E)**, isolates were tested on chrome azurol S agar plates; and yellow halo surrounding the colony indicates the excretion of siderophores. In **(A-E)**, isolates #21, #7 and #11 were considered as auxin-, polyamine and ACCD-producing isolates, respectively. In **(D & E)**, isolates #21 and #7 were considered as P-solubilizing isolates, whereas #21 was considered as a siderophore-producing isolate. Actinobacterial strain #32 was used as a control isolate. IAA, indole-3-acetic acid; Put, putrescine; ACC, 1-aminocyclopropane-1-carboxylic; ACCD, 1-aminocyclopropane-1-carboxylic deaminase; P, phosphorus.

.

Table S1. Comparison of morphological, cultural and phenotypic characteristics that distinguish auxin-producing isolate #21 from very closely related species *Streptomyces chartreusis.*

Characteristics	Isolate #21	*S. chartreusis*
**Morphological**		
Spore chain	Spirales (open spiral) having 10-25 spores/chain.	Spirales (open spiral) having 10-50 spores/chain.
Spore surface	Spiny	Spiny
**Cultural**		
**ISP3**		
Aerial mass color	Powdery blue-green	Powdery blue-green
Substrate mycelium color	Yellowish white	Yellowish white
Production of soluble pigment	No distinctive pigments	No distinctive pigments
**Phenotypic**		
Growth on carbon sources		
Xylose	+	+
Arabinose	+	+
Glucose	+	+
Fructose	+	+
Rhamnose	+	+
Iso-inositol	+	+
Mannitol	+	+
Sucrose	+	+
Raffinose	-	-
Melanin production in		
Peptone yeast iron agar	+	+
Tyrosine agar	-	-
Citrate utilization	+	+
Nitrate reduction	+	+
Hydrolysis of		
Starch	+	+
Gelatin	+	+
Casein	+	+
Production of hydrogen sulfide	-	-
**Reference**	This study	Leach et al. (1953)

+, growth or positive reaction; -, no growth or negative reaction

Table S2. Comparison of morphological, cultural and phenotypic characteristics that distinguish polyamine-producing isolate #7 from very closely related species *Streptomyces tritolerans* and *S. tendae.*

Characteristics	Isolate #7	*S. tritolerans*	*S. tendae*
**Morphological**			
Spore chains	Straight to flexuous (Rectus-flexibilis)	Straight to flexuous (Rectus-flexibilis)	Retinaculiperti-spirals
Spore surface	Smooth	Smooth	warty
**Cultural**			
**ISP3**			
Aerial mycelium color	Gray	Gray	Gray
Substrate mycelium color	Dark yellow	Dark yellow	Yellow
Diffusible pigment color	-	-	Yellow
Growth at 7% NaCl	+	+	-
**Phenotypic**			
Growth on sole carbon sources			
Glucose	+	+	+
Rhamnose	-	-	+
Sucrose	+	+	+
Mannitol	+	+	+
Meso-inositol	-	-	+
Raffinose	-	-	-
Production of melanin pigments on			
Peptone-yeast extract-iron agar	+	+	-
Tyrosine agar	+	+	-
Hydrolysis of			
Casein	+	+	-
Cellulose	+	+	-
Pectin	-	-	+
Starch	+	+	+
Growth on sole nitrogen sources			
L- phenylalanine	+	+	+
L-histidine	-	-	-
**Reference**	This study	Syed et al. (2007)	Ettlinger et al. (1958)

+, growth or positive reaction; -, no growth or negative reaction.

Table S3. Comparison of morphological, cultural and phenotypic characteristics that distinguish 1-aminocyclopropane-1-carboxylic deaminase-producing isolate #11 from very closely related species *Streptomyces rochei, S. plicatus, S. enissocaesilis*, and *S. vinaceusdrappus.*

Characteristic	Isolate #11	*S. rochei*	*S. plicatus*	*S. enissocaesilis*	*S. vinaceusdrappus*
**Morphological**					
Spore chain	Straight to flexuous (Rectus-flexibilis)	Straight to flexuous (Rectus-flexibilis)	Retinaculum-apertum	Spiral	Spiral
Spore surface	Smooth	Smooth	Smooth	Smooth	Smooth
**Cultural**					
**ISP3**					
Aerial mass color	Light grayish yellow	Grayish yellow	Light brownish gray	Poorly developed	Grayish reddish brown
Substrate mycelium color	Pale grayish yellow	Pale grayish yellow	Pale grayish yellow	Brown	Yellow
Diffusible pigment	-	-	-	Brown	-
**ISP4**					
Aerial mass color	Dark grayish yellow	Dark grayish yellow	Light grayish reddish brown	- or poorly developed	Grayish yellowish pink
Substrate mycelium color	Pale grayish yellow	Grayish yellow	Yellowish brown	Colorless	Yellowish brown
Diffusible pigment	-	-	-	-	-
**ISP5**					
Aerial mass color	Light grayish yellow	Light grayish yellow	Light grayish reddish brown	Absent or poorly developed	Grayish reddish brown
Substrate mycelium color	Grayish yellow	Pale grayish yellow	Dark grayish brown	Black grayish brown	Yellowish brown
Diffusible pigment	-	-	-	-	-
**Phenotypic**					
Growth on sole carbon sources					
Glucose	+	+	+	+	+
Arabinose	+	+	+	+	+
Xylose	+	+	+	+	+
Mannitol	+	+	+	+	+
Sucrose	-	-	-	-	+
Rhamnose	+	+	+	-	+
Raffinose	-	-	-	-	+
Fructose	+	+	+	+	+
Production of melanin pigments on					
Peptone-yeast extract-iron agar	-	-	-	-	-
Tyrosine agar	-	-	-	-	-
Production of hydrogen sulfide	-	-	-	-	-
Nitrate reduction	-	-	-	-	-
Hydrolysis of					
Starch	+	+	+	+	+
Gelatin	+	+	+	+	+
Casein	-	-	-	-	-
**Reference**	This study	Kämpfer (2012)	Kämpfer (2012)	Kämpfer (2012)	Kämpfer (2012)

+, growth or positive reaction; -, no growth or negative reaction

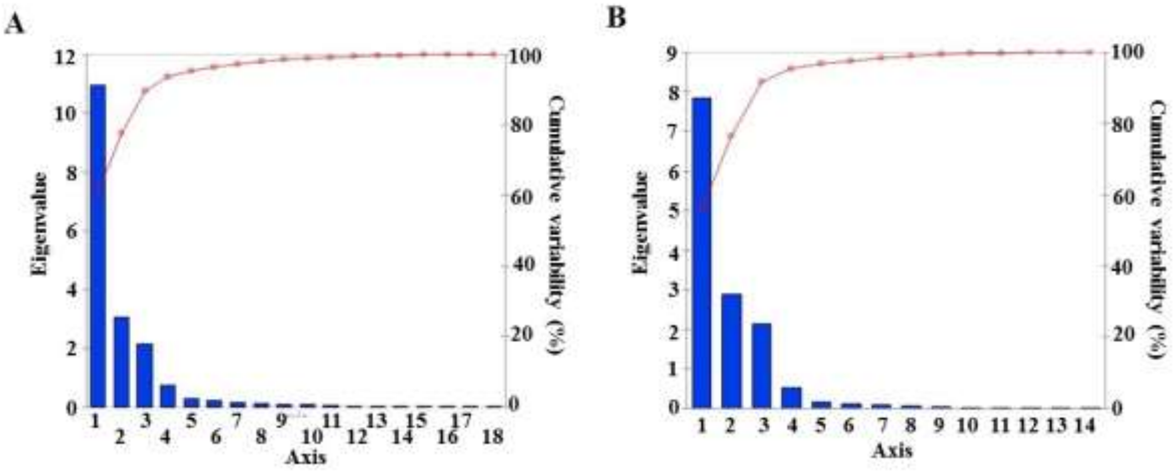

Figure S3. Scree plots representing the eigenvalue of variance accounted for each principal component in **(A)** shoot and **(B)** root. *S. bigelovii* seedlings were grown in an evaporative-cooled greenhouse and maintained at 25±2°C.

Table S4. Pearson correlation coefficient (*n*=8) of 14 variables, including two actinobacterial auxins, six agronomic traits and six plant growth regulators obtained from shoots of *S. bigelovii*. Seedlings were seed- and soil-inoculated with *Streptomyces chartreusis* (*Sc*) grown in an evaporative-cooled greenhouse and maintained at 25±2°C (*, *P*<0.05, **, *P*<0.01 and ***, *P*<0.001).

Parameter		*Sc*		Shoot											
		IAA	IPYA	L	DW	SWD	Chla	Chlb	Car	IAA	IPYA	Put	Spd	Spm	ACC
Sc	IAA	1													
	IPYA	0.996 ***	1												
Shoot	L	0.842 ***	0.842 ***	1											
	DW	0.793 ***	0.778 ***	0.747 ***	1										
	SDW	0.891 ***	0.896 ***	0.693 **	0.830 ***	1									
	Chla	0.675 **	0.693 **	0.561 *	0.597 *	0.675 **	1								
	Chlb	0.870 ***	0.868 ***	0.805 ***	0.745 ***	0.670 **	0.536	1							
	Car	0.797 ***	0.806 ***	0.810 ***	0.633 **	0.701 **	0.636 **	0.757 ***	1						
	IAA	0.829 ***	0.835 ***	0.760 ***	0.685 **	0.737 **	0.688 **	0.866 ***	0.773 ***	1					
	IPYA	0.835 ***	0.849 ***	0.853 ***	0.581 *	0.690 *	0.588 *	0.816 ***	0.886 ***	0.751 ***	1				
	Put	0.262	0.290	0.496	0.388	0.154	0.392	0.497	0.335	0.359	0.496	1			
	Spd	0.313	0.309	0.274	0.417	0.263	0.104	0.234	0.027	0.049	-0.048	0.148	1		
	Spm	0.317	0.335	0.269	0.171	0.191	0.346	0.380	0.689 **	0.335	0.481	0.208	-0.090	1	
	ACC	0.040	0.010	0.254	0.294	0.014	-0.112	0.259	0.021	0.199	0.099	0.335	-0.043	-0.202	1

Table S5. Pearson correlation coefficient (*n*=8) of 10 variables, including two actinobacterial auxins, two agronomic traits and six plant growth regulators obtained from roots of *S. bigelovii*. Seedlings were seed- and soil-inoculated with *Streptomyces chartreusis* (*Sc*) grown in an evaporative-cooled greenhouse and maintained at 25±2°C (*, *P*<0.05, **, *P*<0.01 and ***, *P*<0.001).

Parameter		*Sc*		Root							
		IAA	IPYA	L	DW	IAA	IPYA	Put	Spd	Spm	ACC
Sc	IAA	1									
	IPYA	0.996 ***	1								
Root	L	0.693 **	0.715 **	1							
	DW	0.816 ***	0.847 ***	0.766 ***	1						
	IAA	0.715 **	0.703 **	0.651 **	0.714 **	1					
	IPYA	0.866 ***	0.852 ***	0.465	0.713 **	0.612 *	1				
	Put	0.144	0.182	0.484	0.473	0.221	0.043	1			
	Spd	-0.402	-0.425	-0.013	-0.387	0.041	-0.538 *	-0.083	1		
	Spm	0.344	0.338	0.516 *	0.452	0.743 ***	0.152	0.029	0.349	1	
	ACC	-0.154	-0.141	-0.132	-0.118	-0.391	-0.066	-0.245	-0.310	-0.323	1

Table S6. Pearson correlation coefficient (*n*=8) of 15 variables, including three actinobacterial polyamines, six agronomic traits and six plant growth regulators obtained from shoots of *S. bigelovii*. Seedlings were seed- and soil-inoculated with *Streptomyces tritolerans* (*St*) grown in an evaporative-cooled greenhouse and maintained at 25±2°C (*, *P*<0.05, **, *P*<0.01 and ***, *P*<0.001).

Parameter		*St*			Shoot											
		Put	Spd	Spm	L	DW	SWD	Chla	Chlb	Car	IAA	IPYA	Put	Spd	Spm	ACC
St	Put	1														
	Spd	0.986 ***	1													
	Spm	0.952 ***	0.934 ***	1												
Shoot	L	0.957 ***	0.961 ***	0.919 ***	1											
	DW	0.916 ***	0.896 ***	0.850 ***	0.890 ***	1										
	SDW	0.944 ***	0.924 ***	0.942 ***	0.921 ***	0.901 ***	1									
	Chla	0.891 ***	0.884 ***	0.830 ***	0.840 ***	0.825 ***	0.858 ***	1								
	Chlb	0.948 ***	0.926 ***	0.930 ***	0.942 ***	0.863 ***	0.934 ***	0.848 ***	1							
	Car	0.906 ***	0.889 ***	0.911 ***	0.893 ***	0.786 ***	0.917 ***	0.812 ***	0.936 ***	1						
	IAA	0.851 ***	0.844 ***	0.757 ***	0.743 ***	0.722 **	0.781 ***	0.810 ***	0.755 ***	0.767 ***	1					
	IPYA	0.789 ***	0.804 ***	0.773 ***	0.794 ***	0.679 **	0.748 ***	0.670 **	0.828 ***	0.778 ***	0.529 *	1				
	Put	0.977 ***	0.964 ***	0.923 ***	0.961 ***	0.897 ***	0.948 ***	0.888 ***	0.964 ***	0.937 ***	0.804 ***	0.828 ***	1			
	Spd	0.976 ***	0.961 ***	0.966 ***	0.960 ***	0.891 ***	0.915 ***	0.856 ***	0.918 ***	0.887 r***	0.802 ***	0.747 ***	0.941 ***	1		
	Spm	0.943 ***	0.923 ***	0.911 ***	0.895 ***	0.823 ***	0.884 ***	0.850 ***	0.939 ***	0.958 ***	0.828 ***	0.825 ***	0.947 ***	0.918 ***	1	
	ACC	-0.155	-0.174	-0.184	-0.240	-0.036	-0.266	-0.287	-0.346	-0.382	-0.258	-0.176	-0.248	-0.104	-0.263	1

Table S7. Pearson correlation coefficient (*n*=8) of 11 variables, including three actinobacterial polyamines, two agronomic traits and six plant growth regulators obtained from roots of *S. bigelovii*. Seedlings were seed- and soil-inoculated with *Streptomyces tritolerans* (*St*) grown in an evaporative-cooled greenhouse and maintained at 25±2°C (*, *P*<0.05, **, *P*<0.01 and ***, *P*<0.001).

Parameter		*St*			Root							
		Put	Spd	Spm	L	DW	IAA	IPYA	Put	Spd	Spm	ACC
St	Put	1										
	Spd	0.986***	1									
	Spm	0.952***	0.934***	1								
Root	L	0.922***	0.905***	0.949***	1							
	DW	0.926***	0.918***	0.948***	0.848***	1						
	IAA	0.886***	0.890***	0.871***	0.840***	0.879***	1					
	IPYA	0.790***	0.843***	0.724**	0.619*	0.818***	0.719**	1				
	Put	0.977***	0.967***	0.962***	0.929***	0.959***	0.888***	0.787***	1			
	Spd	0.962***	0.954***	0.925***	0.935***	0.914***	0.897***	0.778***	0.969***	1		
	Spm	0.943***	0.918***	0.915***	0.898***	0.862***	0.900***	0.700**	0.892***	0.917***	1	
	ACC	0.247	0.209	0.192	0.241	0.095	0.163	0.006	0.210	0.168	0.312	1

Table S8. Pearson correlation coefficient (*n*=8) of 13 variables, including the actinobacterial 1-aminocyclopropane-1-carboxylic deaminase , six agronomic traits and six plant growth regulators obtained from shoots of *S. bigelovii*. Seedlings were seed- and soil-inoculated with *Streptomyces rochei* (*Sr*) grown in an evaporative-cooled greenhouse and maintained at 25±2°C (*, *P*<0.05, **, *P*<0.01 and ***, *P*<0.001).

Parameter		*Sr*	Shoot											
		ACCD	L	DW	SWD	Chla	Chlb	Car	IAA	IPYA	Put	Spd	Spm	ACC
Sr	ACCD	1												
Shoot	L	0.889 ***	1											
	DW	0.978 ***	0.893 ***	1										
	SDW	0.959 ***	0.827 ***	0.970 ***	1									
	Chla	0.726 **	0.520 *	0.745 ***	0.724 **	1								
	Chlb	0.964 ***	0.871 ***	0.915 ***	0.880 ***	0.655 **	1							
	Car	0.974 ***	0.845 ***	0.968 ***	0.953 ***	0.729 **	0.949 ***	1						
	IAA	0.943 ***	0.774 ***	0.884 ***	0.908 ***	0.728 **	0.900 ***	0.922 ***	1					
	IPYA	0.863 ***	0.763 ***	0.830 ***	0.830 ***	0.552 *	0.888 ***	0.825 ***	0.753 ***	1				
	Put	0.565 *	0.607 *	0.573 *	0.470	0.445	0.616 *	0.568 *	0.488	0.560 *	1			
	Spd	-0.088	0.158	-0.063	-0.213	-0.171	-0.051	-0.128	-0.178	-0.171	0.222	1		
	Spm	0.179	-0.054	0.186	0.248	0.300	0.199	0.334	0.275	0.192	0.024	-0.213	1	
	ACC	-0.965 ***	-0.820 ***	-0.923 ***	-0.934 ***	-0.753 ***	-0.931 ***	-0.954 ***	-0.956 ***	-0.820 ***	-0.471	0.181	-0.302	1

Table S9. Pearson correlation coefficient (*n*=8) of nine variables, including the the actinobacterial 1-aminocyclopropane-1-carboxylic deaminase, two agronomic traits and six plant growth regulators obtained from roots of *S. bigelovii*. Seedlings were seed- and soil-inoculated with *Streptomyces rochei* (*Sr*) grown in an evaporative-cooled greenhouse and maintained at 25±2°C (*, *P*<0.05, **, *P*<0.01 and ***, *P*<0.001).

Parameter		*Sr*	Root							
		ACCD	L	DW	IAA	IPYA	Put	Spd	Spm	ACC
Sr	ACCD	1								
Root	L	0.701 **	1							
	DW	0.848 ***	0.766 ***	1						
	IAA	0.710 **	0.651 **	0.714 **	1					
	IPYA	0.930 ***	0.465	0.713 **	0.612 *	1				
	Put	0.238	0.484	0.473	0.221	0.043	1			
	Spd	-0.472	-0.013	-0.387	0.041	-0.538 *	-0.083	1		
	Spm	0.269	0.516 *	0.452	0.743 ***	0.152	0.029	0.349	1	
	ACC	-0.114	-0.132	-0.118	-0.391	-0.066	-0.245	-0.310	-0.323	1

Table S10. Pearson correlation coefficient (*n*=8) of 18 variables, including actinobacterial auxins, polyamines and 1-aminocyclopropane-1-carboxylic deaminase, six agronomic traits and six plant growth regulators obtained from shoots of *S. bigelovii*. Seedlings were seed- and soil-inoculated with *S. chartreusis*, *S. tritolerans* and *S. rochei* (*Sc*/*St*/*Sr*) grown in an evaporative-cooled greenhouse and maintained at 25±2°C (*, *P*<0.05, **, *P*<0.01 and ***, *P*<0.001).

Parameter		*Sc*/*St*/*Sr*						Shoot											
		IAA	IPYA	Put	Spd	Spm	ACCD	L	DW	SWD	Chla	Chlb	Car	IAA	IPYA	Put	Spd	Spm	ACC
Sc/*St*/*Sr*	IAA	1																	
	IPYA	0.995***	1																
	Put	0.955***	0.962***	1															
	Spd	0.948***	0.950***	0.975***	1														
	Spm	0.907***	0.898***	0.944***	0.907***	1													
	ACCD	0.949***	0.952***	0.998***	0.979***	0.945***	1												
Shoot	L	0.923***	0.934***	0.971***	0.937***	0.951***	0.970***	1											
	DW	0.949***	0.947***	0.978***	0.952***	0.971***	0.978***	0.981***	1										
	SDW	0.957***	0.939***	0.957***	0.942***	0.952***	0.963***	0.940***	0.975***	1									
	Chla	0.891***	0.904***	0.935***	0.928***	0.908***	0.934***	0.912***	0.933***	0.893***	1								
	Chlb	0.964***	0.957***	0.973***	0.970***	0.962***	0.976***	0.959***	0.975***	0.976***	0.916***	1							
	Car	0.920***	0.911***	0.969***	0.944***	0.982***	0.973***	0.953***	0.978***	0.964***	0.917***	0.974***	1						
	IAA	0.857***	0.864***	0.948***	0.944***	0.913***	0.954***	0.912***	0.916***	0.899***	0.930***	0.918***	0.943***	1					
	IPYA	0.607	0.599	0.684**	0.595	0.691**	0.666**	0.636**	0.671**	0.638**	0.555*	0.634**	0.695**	0.615*	1				
	Put	0.946***	0.961***	0.980***	0.975***	0.912***	0.977***	0.958***	0.957***	0.917***	0.950***	0.950***	0.932***	0.934***	0.629**	1			
	Spd	0.925***	0.921***	0.978***	0.960***	0.921***	0.982***	0.958***	0.963***	0.948***	0.897***	0.954***	0.962***	0.935***	0.673**	0.955***	1		
	Spm	0.934***	0.935***	0.943***	0.955***	0.904***	0.939***	0.892***	0.919***	0.890***	0.918***	0.936***	0.936***	0.917***	0.622*	0.956***	0.920***	1	
	ACC	-0.824***	-0.852***	-0.913***	-0.898***	-0.770***	-0.906***	-0.836***	-0.823***	-0.785***	-0.857***	-0.829***	-0.834***	-0.904***	-0.600*	-0.905***	-0.889***	-0.879***	1

Table S11. Pearson correlation coefficient (*n*=8) of 14 variables, including actinobacterial auxins, polyamines and 1-aminocyclopropane-1-carboxylic deaminase, two agronomic traits and six plant growth regulators obtained from roots of *S. bigelovii*. Seedlings were seed- and soil-inoculated with *S. chartreusis*, *S. tritolerans* and *S. rochei* (*Sc*/*St*/*Sr*) grown in an evaporative-cooled greenhouse and maintained at 25±2°C (*, *P*<0.05, **, *P*<0.01 and ***, *P*<0.001).

Parameter		*Sc*/*St*/*Sr*						Root							
		IAA	IPYA	Put	Spd	Spm	ACCD	L	DW	IAA	IPYA	Put	Spd	Spm	ACC
Sc/*St*/*Sr*	IAA	1													
	IPYA	0.995 ***	1												
	Put	0.955 ***	0.962 ***	1											
	Spd	0.948 ***	0.950 ***	0.975 ***	1										
	Spm	0.907 ***	0.898 ***	0.944 ***	0.907 ***	1									
	ACCD	0.949 ***	0.952 ***	0.998 ***	0.979 ***	0.945 ***	1								
Root	L	0.929 ***	0.932 ***	0.973 ***	0.962 ***	0.954 ***	0.976 ***	1							
	DW	0.962 ***	0.970 ***	0.979 ***	0.968 ***	0.957 ***	0.975 ***	0.960 ***	1						
	IAA	0.891 ***	0.913 ***	0.943 ***	0.943 ***	0.852 ***	0.943 ***	0.897 ***	0.947 ***	1					
	IPYA	0.779 ***	0.799 ***	0.800 ***	0.837 ***	0.664 **	0.787 ***	0.728 **	0.799 ***	0.781 ***	1				
	Put	0.918 ***	0.927 ***	0.975 ***	0.948 ***	0.959 ***	0.974 ***	0.982 ***	0.968 ***	0.901 ***	0.757 ***	1			
	Spd	0.944 ***	0.957 ***	0.961 ***	0.970 ***	0.915 ***	0.958 ***	0.972 ***	0.968 ***	0.915 ***	0.789 ***	0.962 ***	1		
	Spm	0.917 ***	0.909 ***	0.946 ***	0.908 ***	0.887 ***	0.944 ***	0.895 ***	0.908 ***	0.899 ***	0.713 **	0.873 ***	0.864 ***	1	
	ACC	-0.928 ***	-0.930 ***	-0.967 ***	-0.978 ***	-0.905 ***	-0.974 ***	-0.948 ***	-0.956 ***	-0.939 ***	-0.801 ***	-0.941 ***	-0.950 ***	-0.905 ***	1
